# Stem cell- derived extracellular vesicles as new tools in regenerative medicine - Immunomodulatory role and future perspectives

**DOI:** 10.3389/fimmu.2023.1120175

**Published:** 2023-01-24

**Authors:** Elżbieta Karnas, Patrycja Dudek, Ewa K. Zuba-Surma

**Affiliations:** Department of Cell Biology, Faculty of Biochemistry, Biophysics and Biotechnology, Jagiellonian University, Krakow, Poland

**Keywords:** extracellular vesicles, stem cells, paracrine activity, immunomodulation, inflammation, regenerative medicine, tissue injury

## Abstract

In the last few decades, the practical use of stem cells (SCs) in the clinic has attracted significant attention in the regenerative medicine due to the ability of these cells to proliferate and differentiate into other cell types. However, recent findings have demonstrated that the therapeutic capacity of SCs may also be mediated by their ability to secrete biologically active factors, including extracellular vesicles (EVs). Such submicron circular membrane-enveloped vesicles may be released from the cell surface and harbour bioactive cargo in the form of proteins, lipids, mRNA, miRNA, and other regulatory factors. Notably, growing evidence has indicated that EVs may transfer their bioactive content into recipient cells and greatly modulate their functional fate. Thus, they have been recently envisioned as a new class of paracrine factors in cell-to-cell communication. Importantly, EVs may modulate the activity of immune system, playing an important role in the regulation of inflammation, exhibiting broad spectrum of the immunomodulatory activity that promotes the transition from pro-inflammatory to pro-regenerative environment in the site of tissue injury. Consequently, growing interest is placed on attempts to utilize EVs in clinical applications of inflammatory-related dysfunctions as potential next-generation therapeutic factors, alternative to cell-based approaches. In this review we will discuss the current knowledge on the biological properties of SC-derived EVs, with special focus on their role in the regulation of inflammatory response. We will also address recent findings on the immunomodulatory and pro-regenerative activity of EVs in several disease models, including *in vitro* and *in vivo* preclinical, as well as clinical studies. Finally, we will highlight the current perspectives and future challenges of emerging EV-based therapeutic strategies of inflammation-related diseases treatment.

## 1 Introduction

Inflammation is one of the essential reactions of the body for the tissue damage that triggers a cascade of events accompanying the recruitment of immune cells into the site of injury. However, dysregulation or overactivation of the immune system may lead to the several pathological conditions such as life-threatening cytokine storm, fibrosis, uncontrolled infections, autoimmune diseases or cancer (1).

Tissue regeneration is one of the most dynamically developing fields of the contemporary medical sciences, that also includes the development of strategies that would effectively modulate inflammatory response, reducing harmful pro-inflammatory phenotype and promoting reparatory mechanisms. The pivotal role in this area is played by the stem cell-based therapeutic strategies, that take an advantage from the unique features of those cells including self-renewal and differentiation capacity, that may be critical for their successful use in the translational medicine. However, recent years of studies have revealed that SCs may contribute to the tissue repair and immunomodulation of the local environment by several different pathways, mainly those mediated by their secretory activity that also includes release of the biologically active extracellular vesicles (EVs). Indeed, growing data demonstrate that SC-derived EVs (SCs-EVs) may serve as potential new-generation cell-free therapeutic agents that share similar biological features with their cells of origin (2). Many studies indicate, that EVs may not only regulate the crosstalk between innate and adaptive immune system, but most importantly, they may be important players in the treatment of inflammation-related disorders, exhibiting immunomodulatory and pro-regenerative activity, contributing to the restoration of homeostasis (3).

## 2 EVs as paracrine factors with diverse biological functions

### 2.1 Definition and classification of EVs

Extracellular vesicles (EVs) are a heterogeneous population of membrane-enclosed vesicles that are released from the cell surface and possess no ability to replicate (4). EVs are secreted by both normal cells, as well as neoplastic and apoptotic cells, and their presence has also been found in several body fluids, including saliva, urine, milk or amniotic fluid (5). For several years the classification of EVs was based on their size and the cellular compartment of their origin, which also influences their different molecular composition. Thus, three main groups of EVs have been initially recognized: exosomes, ectosomes, apoptotic bodies and oncosomes (6).

Exosomes are considered as a group of vesicles ranging in size from about 30 nm to 120 nm. They are secreted by exocytosis as a result of the fusion of multivesicular bodies (MVBs) with the cell membrane, which results in the release of cargo-containing exosomes into the extracellular area. As exosomes are formed in the late endosomal compartment, they are believed to be enriched in proteins from the tetraspanin (CD9, CD63, CD81) and heat shock family (HSP70 and HSP90), as well as proteins involved in sorting and endosomal transport, such as e.g. apoptosis-linked gene 2-interacting protein X (Alix) or TSG100 (7). Ectosomes, also called microvesicles, have a diameter of 50 nm to 1 µm and are released from the cell surface by the protrusion of a membrane fragment and disruption of the subcellular cytoskeleton, leading to vesicle formation and its budding from the cell surface. They were demonstrated to be enriched in selectins, integrins, CD40L, phosphatidylserine, and a number of other cell-membrane molecules characteristic for the cells which they are derived from (8). Apoptotic bodies are vesicles ranging in size from 50 nm to 2 µm, that are formed as a result of cell fragmentation during the process of programmed death (apoptosis). The mechanism of their formation leads to the enrichment in histones and phosphatidylserine, but they were also shown to contain DNA fragments as a consequence of their mechanism of formation (9).

Oncosomes are considered as a separate group of EVs that are vesicles secreted by the cancer cells. They are usually larger (1-10 µm) and have tumor markers on their surface. They can be classified as a cell-specific fraction of ectosomes secreted by cancer cells, playing an important role in the interaction with cells present in the tumor microenvironment, including cellular components of the immune system (10).

#### 2.1.2 Challenges in EV nomenclature

Despite the fact that the indicated classification of EVs is still commonly used in the majority of papers, there has been a growing issue related to the collective definition of different vesicular entities that have been reported so far. EVs encompass rapidly developing, but still relatively new field of scientific interest, with constantly evolving knowledge on their biology, accompanied by emerging experimental approaches and newly developed methodologies. Thus, in 2014 International Society for Extracellular Vesicles (ISEV) in its first position paper has initially provided criteria of EV definition, as well as minimal set of methodological standards and appropriate experimental controls that should be taken into the consideration in EV-related studies, to provide accurate data that reliably supports the stated conclusions (11). Later on, following the progress in the field and further verification of previously established guidelines, ISEV released updated position paper in 2018, pointing out the need for further standardization of experimental approaches (4). Nevertheless, growing evidence demonstrates the lack of consensus and equivocal data on unique markers and subcellular origin of particular EV subsets, with several indications on morphological and phenotype characteristics to overlap between different vesicular fractions (12). Additionally, several new EV subtypes were recently reported, including exomeres, exophers, or migrasomes (13), which demonstrates the complexity of cellular secreting machinery. Moreover, ISEV points out growing overuse of term “exosomes” without clear experimental evidence on their identity, which leads to misunderstanding and misinterpretation of inaccurate data (14). It is also challenging to exclusively isolate homogenous fraction of exosomes without other EV subtypes (15). Furthermore, depending on the type and source of the starting material, as well as an isolation method, there may be a significant variation in the composition of obtained EV pools, additionally impacted by the heterogeneity of the reported protocols (16).

Thus, taking into account recent advances in the understanding of EV biology and the development of methodological approaches, recently established new ISEV guidelines recommend to avoid direct categorisation on “exosome” or “microvesicle” terms and to use general term “EVs” instead. Eventually, some operational terms for EV subtypes, that relate to their biophysical properties that have been well characterized experimentally in particular study, such as “small/large EVs”, “CD81+ EVs” etc. may also be applied (4). Thus, in current review we will use general term of “EVs” that collectively combines several types of vesicular particles reported in the cited literature.

### 2.2 Molecular composition of EVs

EVs are well known to contain several types of biomolecules that come from their parental cells. The molecular content of EVs is a consequence of their vesicular structure, where a small fragment of the cytoplasm is surrounded by a lipid bilayer. Thus, the bioactive composition of EVs is mainly determined by the type of cells from which they are derived from, as well as the mechanisms of their formation in the cell. It has been also shown that this content may also depend on the activation state of the cell (17). Currently, thousands of different RNA, proteins and lipids have been identified in EVs and were classified e.g. in the ExoCarta database (18). From a functional point of view, the rich molecular composition of EVs can be transferred from vesicle-producing cells to other target cells, affecting their functional status, which may be utilized to modulate the functions of various cells both *in vitro* and *in vivo*.

EVs contain a lipid components which are mainly a part of the biological membrane surrounding the cytoplasmic part of the vesicle. Despite the typical components of cell membrane that can be found in EV membrane, particular enrichment in a cholesterol, sphingomyelins, phosphatidylcholine and phosphatidylethanolamines has been also shown (19), indicating an important role of those molecules in the process of vesicle segregation in MVBs (20). Additionally, the role of the lipid content was also shown to take a part in the biological activity of EVs (21).

Among the key bioactive components of the cytoplasmic part of the EVs, two basic components can be distinguished, including proteins and nucleic acids. The protein content of EVs is enriched in proteins of the endosomal compartment, including Rab GTPases and SNAP (soluble NSF attachment protein) receptor (SNARE) proteins involved in the fusion of vesicles with the cell membrane, but also annexins, flotilllin, as well as proteins related to EVs biogenesis, e.g. Alix and Tsg101 (22). In addition, EVs are also enriched in the proteins that are a part of membrane microdomains and lipid rafts, including tetraspanins (23). It was also demonstrated that EVs may contain several other regulatory factors such as transcription factors (24), enzymes (25), growth factors (26), cytokines and signaling molecules (27).

EVs also contain nucleic acids, in particular RNA, found mainly in the form of mRNA and miRNA. Importantly, the presence of the latter RNA type, known as an important regulatory molecules, pays particular scientific attention in the context of potential bioactive compounds responsible for the functional activity of EVs (28). Currently, the presence of a mechanism for selective sorting and packing of miRNAs into EVs is postulated, as evidenced by numerous studies showing the enrichment of some miRNAs in vesicles, when compared to their donor cells (29). So far, the detailed mechanism of such selectivity is still not fully understood. Nevertheless, several concepts have been proposed, including the role of RNA-induced silencing (RISC) complex, involved in the binding of miRNA to proteins from the Argonaute family (30). Other studies have also demonstrated that heterogeneous nuclear ribonucleoprotein A2B1 (hnRNPA2B1) may be responsible for the control of miRNA loading into EVs (31). Interestingly, recent research demonstrates that in addition to mRNA and miRNA, EVs may also contain other types of non-coding RNA, including transporting RNA (tRNA), small interfering RNA (siRNA) or vault RNA (vRNA) (32). However, the risk of non-EV-associated extracellular protein-RNA complexes that may be co-isolated with EV preparations must be always carefully considered in the data interpretation.

Recent studies also indicate the presence of genomic DNA in EVs, which enables its horizontal transfer between cells, resulting in a modulation of gene expression, and thus influencing the biological characteristics of cells. For example, nearly 350 chromosomal DNA sequences have been identified in EVs produced by cardiomyocytes (33). In addition, the presence of mitochondrial DNA has been also demonstrated (34). Similarly to other components of EVs, a certain selectivity of DNA fragments has been also observed as a result of both EV type and the activation state of the secreting cells (35).

### 2.3 EVs biogenesis and secretion

**Figure 1:**
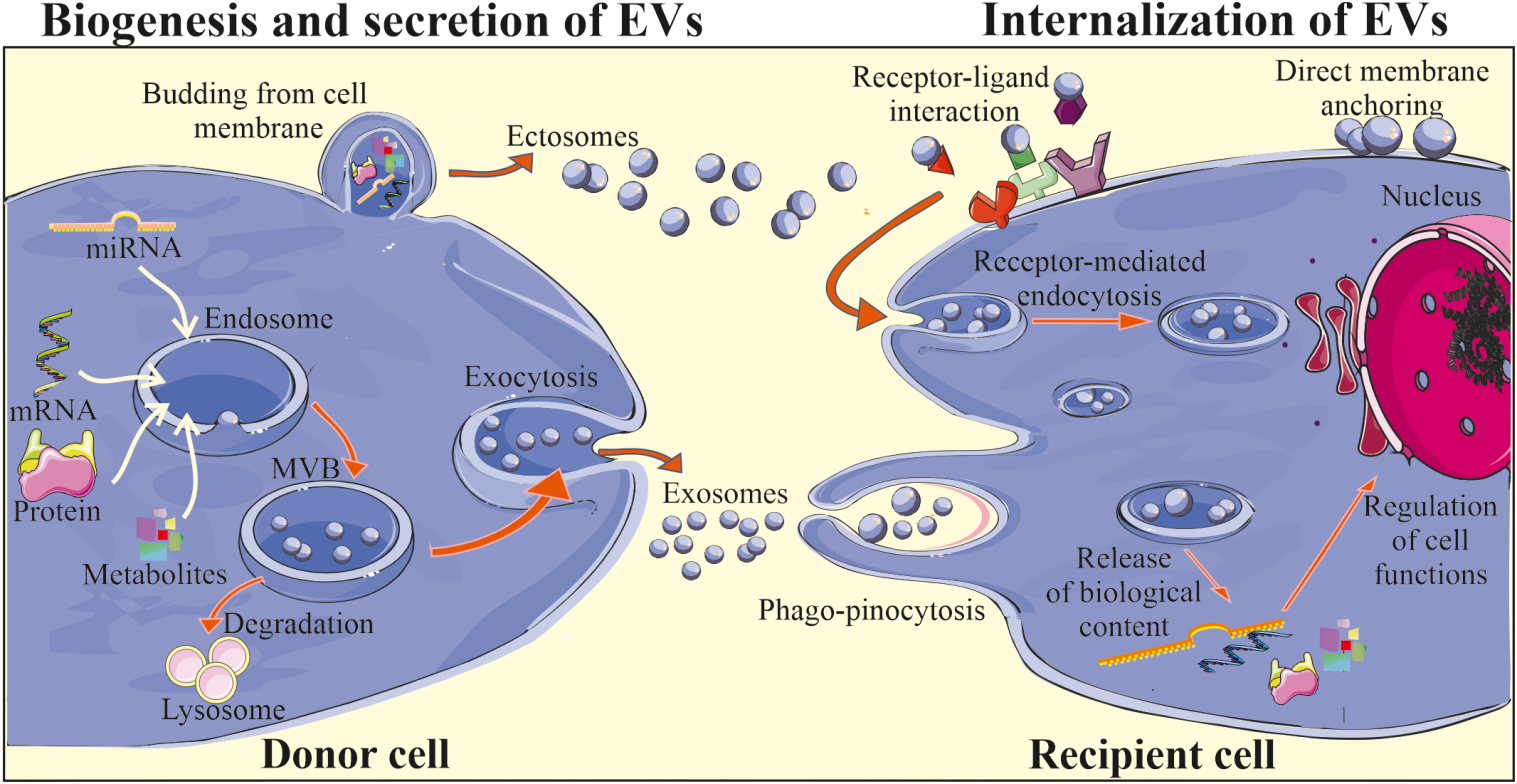
Biogenesis and biological activity of EVs. Two main subtypes of EVs are exosomes and ectosomes (microvesicles) that differ in terms of their biogenesis and secretion. Exosomes are initially formed in MVBs located in the cytoplasm, with the involvement of endosomal pathway and intracellular trafficking of MVBs, that may be either degraded in the lysosomes or may fuse with the plasma membrane, releasing exosomes into the extracellular milieu. Ectosomes are considered to be generally larger than exosomes and are formed through the outward plasma membrane budding and shedding. After release EVs may interact with the recipient cells, delivering their cargo *via* direct fusion with cell membrane, endocytosis, receptor-ligand interaction or phago/pinocytosis. Consequently, internalization of EV content may lead to the changes in the biological activity of the target cell.

The mechanisms of biogenesis and secretion may vary depending on a type of EVs (**Figure 1**). Exosomes are considered to be initially formed in MVBs, which may be either degraded upon association with lysosomes or may be secreted by exocytosis (36). The two-way fate of the MVBs may be determined by their lipid content, where cholesterol-rich MVB populations have been shown to be secreted (37) and lysobisphosphatidic acid- enriched ones to bind to lysosomes and be degraded (38). The formation of MVBs involves the segregation of their contents at the endosome’s boundary membrane and the subsequent budding of intraluminal vesicles into its interior. This process involves endosomal sorting complex responsible for transport (ESCRT) associated with Alix proteins and syntenin (39). However, some studies suggest that MVBs formation may also occur independently of ESCRT complexes, with the simultaneous involvement of sphingomyelinases that enrich exosomes in ceramides (40). The participation of tetraspanins in exosome formation was also demonstrated (41). In the case of ectosomes, the mechanism of their formation is less understood. Nevertheless, it has been shown that their formation is accompanied by oligomerization of cytoplasmic proteins and their anchoring in the cell membrane by myristoylation and palmitoylation (42). The participation of the actin cytoskeleton and proteins from the GTPase family in the ectosome formation process has also been reported (43).

The exact mechanism of EV release from the cell surface is still not fully revealed. However, it has been shown to be accompanied by the reorganization of the sub-membranous cytoskeleton and involvement of Rab GTPases and SNARE proteins, that are responsible for the fusion of vesicles with the cell membrane (44). Moreover, it is possible to externally stimulate cells to secrete EVs, e.g. by activating the thrombin receptor in the case of platelets (45), inducing an increase in the intracellular calcium ions concentration (46) or stimulation of dendritic cells (DCs) by the lipopolysaccharide treatment (47).

### 2.4 Biological activity of EVs

For several years EVs were considered as contaminants and debris lacking an essential biological function. Later on, EVs were envisioned as a waste disposal machinery, which allows cells to rapidly get rid of a molecules and metabolites that are not needed anymore (48). However, in last few decades remarkable advance in the understanding of EV biology have been done together with the growing number of scientific reports confirming an important role of EVs as part of the paracrine activity of cells (49). Indeed, subsequent studies have demonstrated the role of EVs in the process of information exchange between the cells. It has been widely postulated that EVs may contribute to the cell-to-cell communication, which includes the step of their interaction with the target cell, that may occur in several ways: by endocytosis, phagocytosis, or by direct fusion with the cell membrane including receptor-ligand interactions, subsequently leading to the release of bioactive cargo (**Figure 1**) (50). The exact mechanism of EV binding to the cell membrane of recipient cell is still not thoroughly investigated. However, it has been demonstrated that e.g. syncytin that binds to major facilitator superfamily domain 2a (MFSD2a) receptors present in the cell membrane may participate in this process (50). Adhesion molecules, including integrins, lipid rafts and proteins from SNARE and Rab families may also mediate the fusion of EVs with cell membrane (36). Interestingly, some selectivity of EV binding to specific types of target cells has also been demonstrated. For example, EVs secreted by neuroblastoma cells showed affinity to neurons and glial cells, while vesicles from stimulated cortical neurons were endocytosed only by neurons (51). One of the postulated mechanisms of selective binding of EV with recipient cell includes the influence of tetraspanins, which interact with integrins and other anchor proteins, modulating their functions (52). Moreover, ligand-receptor interplay may also be involved in the control of this process, as was shown for EVs secreted by endothelial progenitor cells (EPCs) that were reported to bind *via* the C-X-C motif chemokine receptor 4 (CXCR4) to its ligand stromal cell-derived factor 1 (SDF-1) present on the endothelial cells (53).

**Figure 2:**
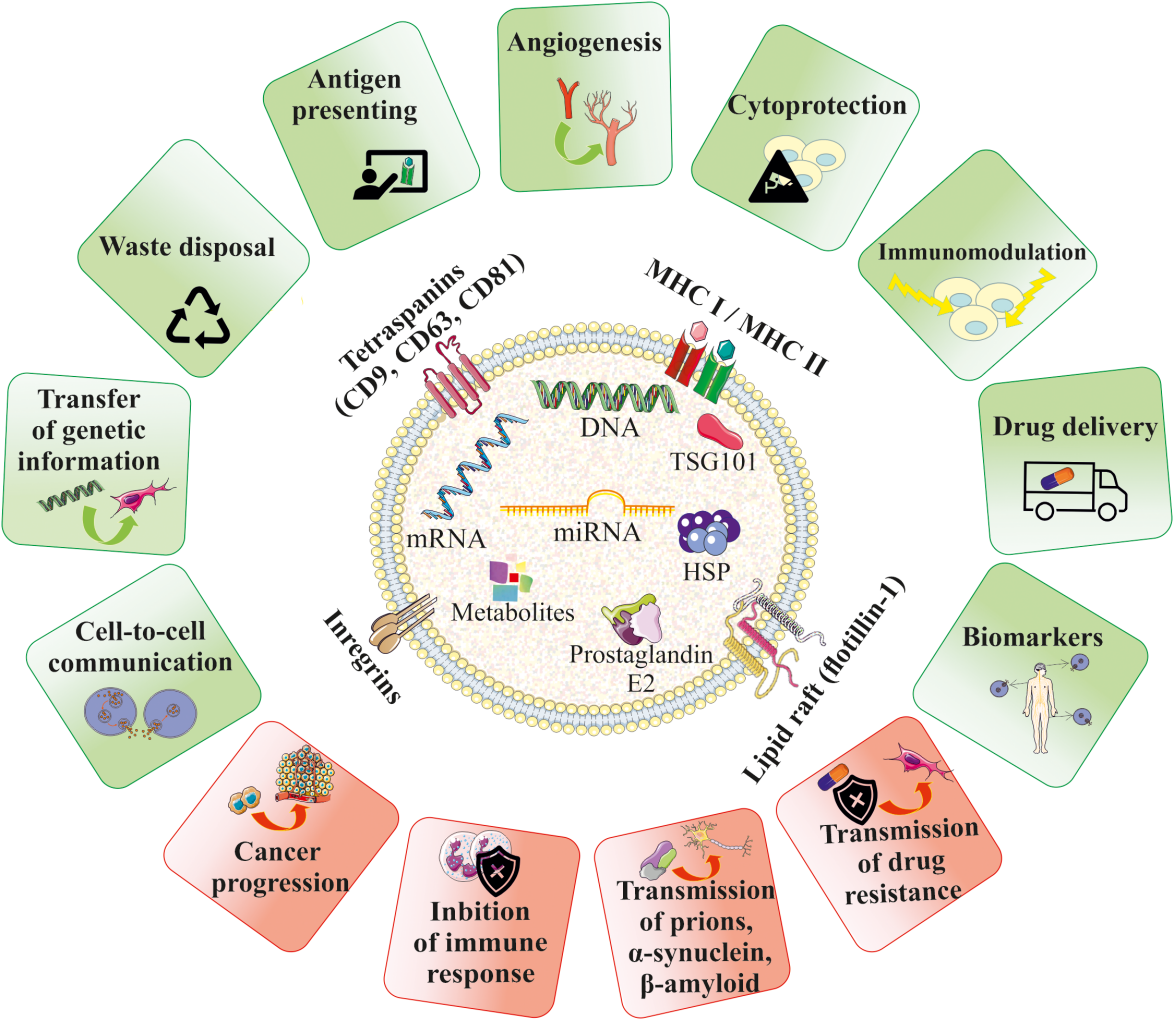
Biological role of EVs in homeostasis and pathophysiology. EVs contain bioactive cargo that is responsible for their multimodal activity. EVs may mimic the properties of their cells of origin and were shown to be paracrine factors that play role in cell-to-cell communication and influence the fate of the target cells in several ways, including e.g. stimulation of angiogenesis, cell survival or modulation of the immune response. EVs may also serve as waste disposal machinery, drug-delivery systems and biomarkers for the diagnostic purposes. An influence of EVs in the development of several diseases has been also reported.

EVs can serve as paracrine mediators that target cells by transferring their bioactive content in the form of different types of nucleic acids, receptors, enzymes, transcription factors, immunomodulators and even morphogenic factors such as Wnt (54) and Notch (55) signaling proteins. Delivery of the EV cargo into the recipient cells opens several ways of potential regulation of cellular processes, including influence on gene and protein expression, as well as activity of intracellular signaling pathways. Depending on the cell origin and the type of the target cells, EVs were showed to either stimulate or inhibit cell proliferation and differentiation, act as cytoprotective agents reducing cell death (56), exert pro-angiogenic stimuli (57), regulate myelin formation (58) and modulate immune cells, as will be discussed below (**Figure 2**). Importantly, EVs may act not only as paracrine factors, transferring the biological information between different types of cells, but were also shown to play pivotal role in the autocrine signaling (59).

On the other hand, EVs may also participate in the pathogenesis of many diseases. As an example, EVs secreted by tumors may promote their progression by stimulation of pro-angiogenic processes and inhibition of the immune system (60). EVs have also been shown to contribute to the transmission of prions (61), α-synuclein responsible for the pathogenesis of Parkinson’s disease (PD) (62), as well as β-amyloid, which contributes to the development of Alzheimer’s disease (AD) (63). Moreover, EVs can transfer the drug resistance phenotype between cells, which is related to the transfer of drug-efflux membrane pumps (**Figure 2**) (64).

### 2.5 Role of EVs in the regulation of immune system

**Figure 3:**
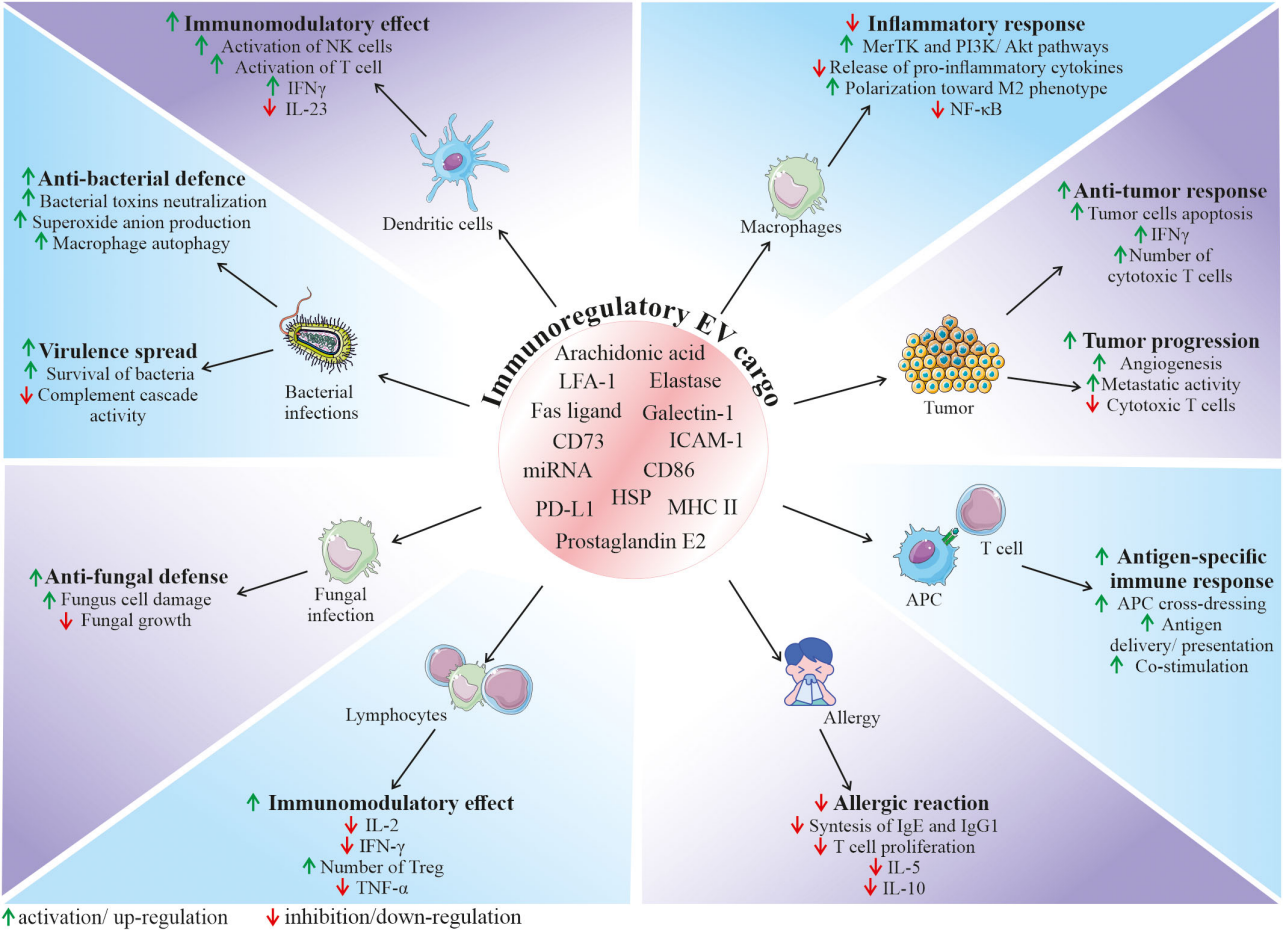
Role of EVs in the regulation of the immune response. Depending on the origin, EVs can contain and deliver a diverse bioactive cargo with immunoregulatory activity, that can influence various cell types and modulate their functional status. It has been demonstrated that EVs may have an impact on many immune-related processes, including regulation of immune system activation status, mediation of anti-bacterial and anti-fungal defence, modulation of anti-tumor response, as well as inhibition of harmful overactivation of the immune system. APC, antigen presenting cells; ICAM-1, intercellular adhesion molecule 1; HSP, heat shock protein; IFN-γ, interferon gamma; LFA-1, lymphocyte function-associated antigen 1; MHC II, major histocompatibility complex class II; MerTK, mer receptor tyrosine kinase; NK cells, natural killer cells; PD-L1, programmed death-ligand 1; TNF-α, tumor necrosis factor alpha; Treg, regulatory T cells.

Among the variety of reported functions, EVs are also envisioned as important factors modulating the function of the immune system, both as activators or inhibitors, depending on the biological context. Their role in the immunity relies both on the interaction of EVs from other cell types with immune cells, as well as on the secretion of EVs by the cellular components of immune system, regulating its fate in the paracrine or autocrine manner (**Figure 3**) (65). Thus, EVs mediate communication between immune cells, taking part in orchestrating an immune response. In particular, they are a part of interaction of innate and adaptive immunity, modulating cell response and release of cytokines, chemokines and other immune-active factors (65).

In the context of immune defence against pathogenic factors, EVs are involved in the communication between bacteria and host cells, playing either protective or pathogenic role in the infection. On one hand, bacteria-derived EVs may serve as a shuttle particles contributing to virulence spread. On contrary, secretion of EVs by the host cells may be a method to expel intracellular bacteria, neutralize bacterial toxins or stimulate both innate and adaptive immune response (66). As an example, EVs secreted by neutrophils infected by *Mycobacterium tuberculosis* stimulated autophagy, expression of costimulatory molecules and superoxide anion production in bacteria-containing macrophages, enhancing their clearance from this intracellular pathogen (67). In another study, EVs produced by DCs infected with *Listeria monocytogenes* stimulated immature DCs to pro-inflammatory state and anti-viral defense (68). An involvement of EVs in the fungal infections has also been demonstrated. For example, it was reported that neutrophils may secrete EVs that act as anti-fungal agents containing antimicrobial cargo such as neutrophil elastase, myeloperoxidase, cathepsin G, azurocidin, and defensin, that may inhibit growth of *Aspergillus fumigatus* (69).

Recent studies put novel insights into the mechanism of EV function in the immune system, which opens new possibilities in the control of immunological response for the therapeutic purposes. Immunoregulatory activity of EVs is related to their biological content, that consist of molecules known to be involved in the regulation of immune cells. As an example, heat shock proteins (HSP) that were shown to be present in EVs are known immunomodulants (70). Several lipid and lipid-related signaling mediators such as phospholipases, prostaglandin E2 or arachidonic acid were also reported to be a part of EV cargo (71). Additionally, presence of major histocompatibility complex (MHC) class II, and co-stimulatory CD86, as well other immunologically-active molecules such lymphocyte function-associated antigen 1 (LFA-1) and intercellular adhesion molecule 1 (ICAM-1) was also shown on EVs derived from antigen presenting cells (APCs), that were able to regulate the proliferation of B and T cells (72). In this context, EVs released by APCs such as macrophages or DCs may participate in the antigen-specific interaction between immune cells *via* the cross-dressing mechanism (65). EVs may bind to the surface of APCs, contributing to the antigen presentation to T cells or may be internalized by APCs, delivering their antigen peptide/MHC complexes, contributing to the antigen spread (73). This mechanism plays a pivotal role in the development of anti-tumor response, where tumor-derived EVs may be taken up by APCs, enhancing cross-presentation of tumor-specific antigens to cytotoxic T cells (74). It has been also shown that EVs secreted by the immune cells can transfer surface Fas ligand on their surface, thereby contributing to the control of cell death during the immune response (75).

Apart from the possible ways of EV-mediated activation of immune system, several findings demonstrate their immunosuppressive role in homeostasis and disease. However, despite growing evidence on multimodal immunomodulation of immune system through EVs, exact mechanism of their action, together with immunomodulatory cargo responsible for this effect still remain to be deeply determined. Nevertheless, several studies have shed light on the potential EV-related factors that may exert their suppressive activity. As an example, EVs secreted by tumor cells were shown to carry programmed death-ligand 1 (PD-L1) that suppresses cytotoxic T cells (76). Additionally, widely postulated immunomodulatory activity of EVs may be an essential mechanism that allows to control excessive or chronic activation of immune system, as well as autoimmunity, thus contributing to protection against several pathological conditions. For instance, neutrophil-derived EVs were shown to inhibit pro-inflammatory cytokine release by macrophages *via* modulation of Mer receptor tyrosine kinase (MerTK) and PI3K/Akt pathways (77), with the possible mechanism of their immunosuppressive action related to the presence of phosphatidylserine (78). miRNA content may be also involved in the immunomodulatory activity of EVs that leads to the anti-inflammatory phenotype of immune cells (79). As an example, EVs from endothelial cells were shown to harbour miR-10a that mediated inhibition of monocyte activation *via* NF-κB pathway, both *in vitro* and *in vivo* (80). The immunomodulatory activity of EVs has also been demonstrated in many other systems, including the respiratory tract, where they decreased allergic reaction (81). In another study, breast milk-derived EVs inhibited activation of peripheral blood mononuclear cells, increasing the number of regulatory T cells (82).

Most importantly, as EVs are natural carriers of several biomolecules that come from their parental cells, they might share functional similarities with their source cells. Thus, the unique biological properties of SCs, including ability to modulate immune system, arouses particular interest in the utilization of their EVs (SCs-EVs) in the context of interaction with the immune system. Indeed, based on the several recent findings, SC-EVs have been recognized and appreciated as a potential mediators inhibiting harmful overactivation of immune cells, accompanied by the simultaneous promotion of beneficial, pro-regenerative phenotype in the site of injury, followed by the restoration of homeostasis (3). Thus, these biological effects of SCs-EVs give a hope to develop new strategies of treatment of several diseases at their various stages. Additionally to the already discussed different types of cargo commonly present in vesicles from different cells, EVs derived from mesenchymal stem cells (MSCs) were shown to contain CD73, which is ecto-5′-nucleotidase capable to convert adenosine monophosphate (AMP) into adenosine, that may bind to A2 receptors present on the surface of immune cells, exerting immunosuppressive effect (83). MSCs-derived EVs (MSCs-EVs) may possess miR-21 that is involved in the activation of tolerogenic transforming growth factor β (TGF-β) signaling (84). Indoleamine 2,3-dioxygenase (IDO) known as a tryptophan-degrading enzyme, transferred in EVs from MSCs and DCs may also mediate their immunomodulatory effect (85, 86). Glycan-binding protein galectin-1 found in EVs from MSCs isolated from placenta is also known as immunomodulatory factor that promotes proliferation of regulatory T cells (Tregs) (87).

Taking together, EVs secreted not only by the immune cells, but also by the SCs may be promising immunoregulatory factors and thus promising candidates for the further development of therapeutic approaches.

## 3 SCs-EVs as an alternative option to cell-based therapies

**Figure 4:**
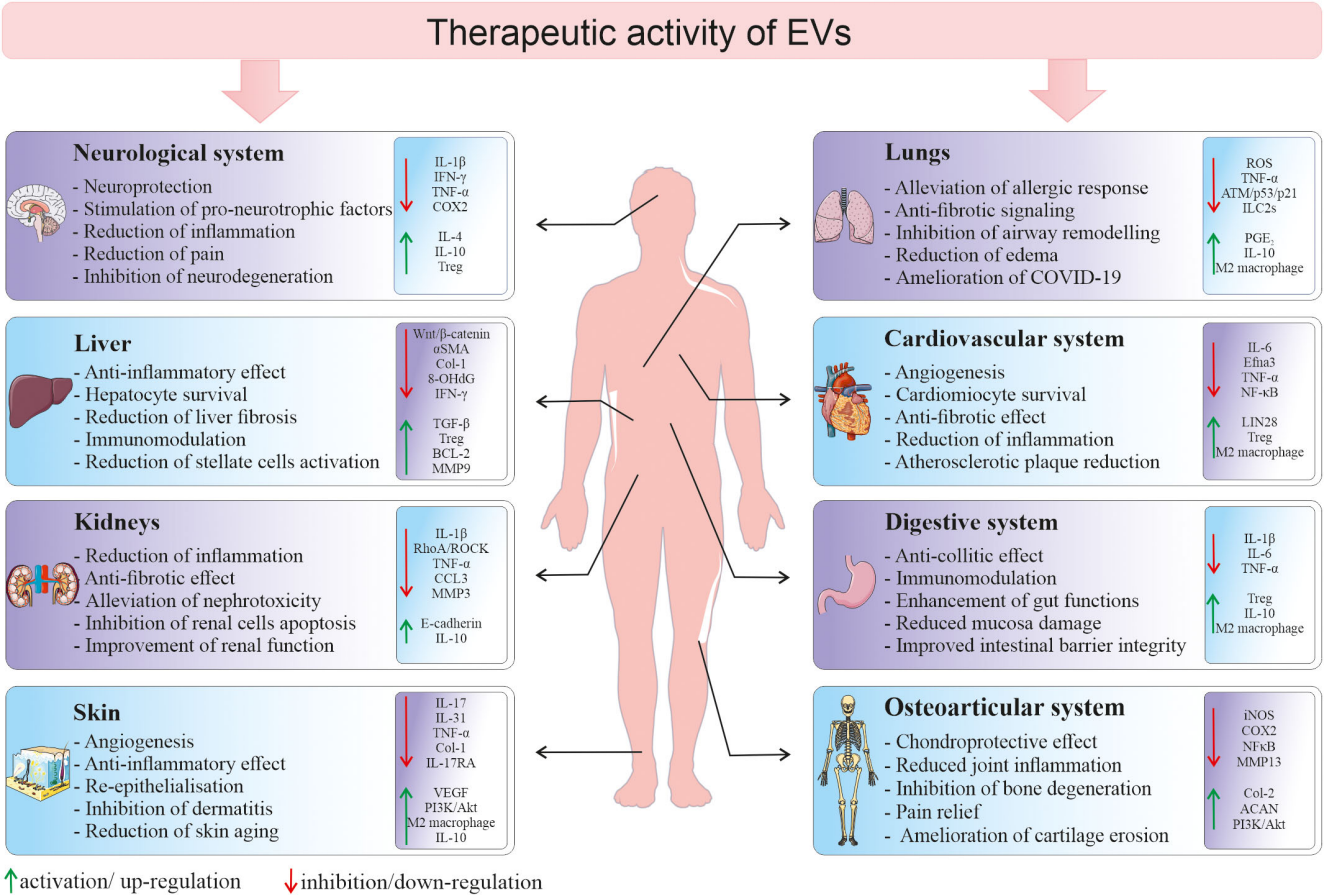
Therapeutic activity of EVs in different tissues and organs. Depending on a type of tissue/organ as a site of vesicle delivery, EVs they may modulate several cellular processes and signaling pathways in the local environment, leading to the tissue regeneration in the place of injury. 8-OHdG, 8-hydroxyguanosine; ACAN, aggrecan; BCL-2, B-cell CLL/lymphoma 2; CCL3, macrophage inflammatory protein-1 α; Col, collagen; COX2, cyclooxygenase-2; Efna3, ephrin A3; IFN-γ, interferon gamma; ILC2s, type 2 innate lymphoid cells; iNOS, inducible nitric oxide synthase; MMP, metalloproteinase; NF-κB, nuclear factor kappa-light-chain-enhancer of activated B cells; PGE_2_, prostaglandin E2; ROS, reactive oxygen species; TGF-β, transforming growth factor β; TNF-α, tumor necrosis factor alpha; Treg, regulatory T cells; VEGF, vascular endothelial growth factor; αSMA, alpha smooth muscle actin.

Due to the increasing evidence that EVs are not only the waste elimination apparatus, but they possess multimodal biological potential, EV field encompass a rapidly growing scientific interest in terms of their possible use in the regenerative medicine. Importantly, they are envisioned as potential new-generation therapeutic tools that may overcome several limitations related to the whole cell-based therapies. Thus, there is growing hope for the use of SCs-EVs as an alternatives to cell therapy, as they may not only mimic the phenotype of the cells from which they originate, but also possess several advantageous features (88). For instance, the utilization of EVs minimizes the risk of developing a tumor resulting from transplanted cells, in particular pluripotent SCs. What is more, direct comparison of the influence of MSCs and their EVs on T-cell subsets proliferation *in vitro*, indicated that the co-culture with MSCs, but not with MSCs-EVs, reduced the proliferation of CD3+ cells. On contrary, EVs stimulated proliferation of Tregs, increased apoptosis of CD3+ cells and elevated level of IL-10. These results may indicate higher immunomodulatory activity of EVs, comparing to their parental cells, which may be beneficial for the therapeutic purposes (89). Moreover, animal studies have shown the potential possibility of administration of EV preparations in the form of aerosols, which allows their local delivery to the respiratory system (endotracheal) or to the central nervous system (intranasally) (90, 91). Additionally, biocompatible lipid bilayer structure of EVs that encloses naturally or exogenously loaded genetic cargo, protecting it against degradation, opens a possibility to use EVs as vectors, that bypass the limitations of virus-based nucleic acid delivery, related to immunogenicity and packaging capacity (92). Importantly, small size of EVs facilitates their transfer throughout the body and enables them to cross blood-brain barrier (BBB) (93). Additionally, there has been an increased interest in the possibility to modify EVs by their engineering that includes either surface or cargo modification, to improve their biological activity or enhance stability and targeted delivery (94). Taking together, the recognition of SCs-EV ability to transfer biologically active molecules between cells and thus their involvement in the paracrine signaling has made them an attractive option for the therapeutic purposes in several experimental models. Importantly, EVs may have a tremendous potential as therapeutic agents for the treatment of several diseases with the inflammatory component (**Figure 4**).

### 3.1 SCs as a source of EVs for the therapeutic applications

The unique ability of SCs to self-renew and differentiate into other types of cells has made them well established and main type of cells for the use in the medicine. For many years the prevailing view was that their regenerative activity is mainly a consequence of the ability to directly rebuild damaged tissue by proliferating and differentiating in the site of injury. However, recent years of research clearly indicate that some of the observed therapeutic effects after SCs administration result from their paracrine activity, related to the secretion of a number of cytokines and growth factors, which stimulate cells residing at the site of damage to undertake reparatory processes (2). Consequently, growing number of reports indicates that SCs, in addition to soluble molecules, may also release bioactive EVs, which may play an essential role in the pro-regenerative activity of those cells (95). Thus, currently several different types of SCs are considered as sources of EVs for the therapeutic applications.

#### 3.1.1 Mesenchymal stem/stromal cells (MSCs)

MSCs are multipotent SCs of mesodermal origin, that are able to differentiate into chondrogenic, osteogenic and adipogenic lineages. They may be isolated from various sources, including bone marrow (BM-MSCs), adipose tissue (AT-MSCs) and postnatal tissues such as umbilical cord (UC-MSCs) (96). MSCs are known for their high secretory activity, which includes release of extracellular matrix (ECM) proteins, cytokines, chemokines, growth factors, but also bioactive EVs that may play a role in mediating crosstalk to local and distant tissues (97). This paracrine activity of MSCs makes them also crucial players in immunomodulation, which may trigger mostly anti-inflammatory signaling and suppress excessive activation of immune system components (98). Importantly, MSCs-EVs were demonstrated to share biological activity with their parental cells, that are known to possess immunomodulatory properties. Several studies have demonstrated an impact of tissue origin on potential differences in the functional activity of MSCs, which may be also reflected in the distinct biological activity of their EVs (99).

MSCs possess limited stemness and differentiation potential and thus reduced risk of teratoma formation when compared to pluripotent SCs. On the other hand, they also have relatively high proliferative capacity *in vitro* and do not require advanced and expensive culture reagents, which allows researchers to effectively reach the level of MSC expansion sufficient for the isolation of EV batches dedicated for the therapeutic applications (100). However, there are still several challenges of the effective use of MSCs as a source of EVs for the therapeutic purposes, including donor variability and need to optimize expansion methods in order to avoid cell senescence. Nevertheless, considering the lack of ethical concerns, ease of isolation from several sources potentially available in both autologous and allogeneic systems, biological safety and low immunogenicity, MSCs have become primary cells of choice for the purpose of the tissue regeneration. A natural consequence of this fact is that researchers are particularly interested in the application of EVs secreted by these cells (101). Thus, numerous studies show that MSC-EVs have significant cytoprotective, regenerative and immunomodulatory potential in several disease models.

#### 3.1.2 Embryonic SCs (ESCs)

ESCs are pluripotent population of cells with unlimited proliferative capacity, capable to give rise into any type of cells within three germ layers. Consequently, ESCs were initially envisioned as potentially ideal type of SCs for the medical purposes (102). However, due to the ethical concerns regarding their sourcing, as well as the risk of teratoma formation, clinical application of ESCs has been highly limited (103). Nevertheless, due to the acellular nature, the use of EVs secreted by already available ESC lines (ESCs-EVs) still remains promising strategy for the regenerative therapies (104). Due to the potentially unlimited quantities of cells, ESCs are often used as starting cells that are differentiated toward more specified progenitors serving as a source of EVs for therapeutic approaches (105). Another interesting approach is to use ESC-EVs to boost the therapeutic efficacy of other SC populations. As an example, ESCs-EVs were demonstrated to reduce senescence and enhance pro-regenerative effects of MSCs in a mouse cutaneous wound model, by activating the PI3K/AKT pathway (106).

#### 3.1.3 Induced pluripotent SCs (iPSCs)

iPSCs were initially obtained by the Prof. Yamanaka’s group by genetic reprogramming of somatic cells through the forced expression of key transcription factors such as Oct3/4, Sox2, Klf4, and c-Myc (107). This achievement was awarded by Nobel Prize in 2012 and has opened new chapter not only in the field of stem cell biology, but also in the area of tissue regeneration. iPSCs display pluripotent properties similar to those of ESCs, allowing relatively easy accessibility to pluripotent cells without ethical problems related to the cells of embryonic origin. Consequently, due to their potentially unlimited proliferative and differentiation potential, iPSCs have been widely used for disease modelling, drug discovery and cell-based therapies, resulting in the substantial progress in the field (108).

Along with their paracrine activity, iPSCs have been also recognized as important donors of EVs for the basic research as well as the therapeutic applications. Similarly to ESCs, iPSCs are also differentiated into other cell types that are sources of EVs for the regenerative purposes (109). Interestingly, iPSCs were shown to be able to secrete EVs more abundantly and with higher capability to enter target cells, when compared to the MSCs (110), which may make these cells advantageous in the context of the donor cells for EV-based therapeutic approaches.

#### 3.1.4 Other SCs types

Despite the special focus on the pluripotent and mesenchymal SC as main sources of EVs for the tissue regeneration, the pro-regenerative potential of EVs secreted by the several other SCs and progenitor cell was also investigated. As an example, therapeutic efficacy of EPCs- derived EVs was shown in different experimental setups (111). Similarly, protective effect of EVs from neural (112) and cardiac progenitors (113) was also demonstrated.

### 3.2 Toward therapeutic applications of SCs-EVs- preclinical studies

The ability of SCs-EVs to modulate immune response indicates that they may be used therapeutically for a broad spectrum of diseases. So far, EVs have been tested in several *in vitro* and *in vivo* preclinical studies that cover a broad range of experimental disease models. In this section we will provide an overview on the different approaches utilized to explore an emerging role of EVs as potential new-generation tools for the tissue and organ regeneration, including their immunoregulatory activity.

#### 3.2.1 Cardiovascular diseases

Cardiovascular diseases (CVDs) are one of the most common causes of death, with limited efficacy of currently available therapeutic strategies. According to the data provided by the world health organization (WHO), CVDs are responsible for about one-third of all death cases worldwide, which corresponds to almost 18 million of human beings every year (114). Cardiac tissue has a limited regenerative capacity and endogenous systems are typically insufficient for the cardiac repair. Once injured, mammalian heart lacks the ability to replace damaged cardiomyocytes, which leads to the progressing loss of its function. Thus, the development of novel therapeutic approaches and identifying intrinsic and external factors together with new potential targets to improve cardiac performance are of special focus (115). CVDs encompass broad spectrum of disorders, but the two major representations of ischemic CVDs are acute myocardial infarction (AMI) and chronic myocardial disease (CMD), which differ in terms of their mechanisms of cause and clinical manifestation, with indispensable role of inflammatory response. Both conditions are life-threatening and lead to the subsequent cardiac remodelling and scar formation rather than regeneration, which can adversely affect function of the cardiovascular system (116).

AMI is a rapid event caused by the coronary artery occlusion by the ruptured plaque that blocks the blood flow, followed by the oxygen deprivation in the myocardium and death of cardiomyocytes. Consequently, due to the insufficient ability of heart to compensate the massive loss of cardiac cells following infarction, injured tissue becomes fibrotic and non-contractile, leading to the heart disfunction such as dilatation, reduced ejection fraction, left ventricle stiffness and its remodelling (117). Current AMI therapeutic strategies include e.g. urgent reperfusion therapy, pharmacotherapy and surgical intervention, including heart transplantation (114). Despite advancement in the treatment, AMI still carries a high mortality rate, with increasing morbidity caused by the several risk factors that are a common part of contemporary, unhealthy lifestyle, such as smoking, obesity, hypertension, lack of physical activity and high exposure to the stress (118). Additionally, patients who survived AMI have a higher risk of recurrent AMI or other CVD-related complications (119). In a consequence, there is a great need for new, more effective therapeutic strategies, including those that would effectively support the natural reparatory mechanisms of the heart muscle and would reduce inflammatory response, minimizing subsequent cardiac tissue deterioration and adverse remodelling.

First attempts in this matter were focused on a cell-based therapies that relied on the administration of several types of stem and progenitors cells, including e.g. BM-MSCs, different populations of cardiac progenitor cells (CPCs) or cardiosphere-derived cells (120–122). However, despite indication on safety and some beneficial effects, the efficacy of cell-based therapies varied depending on a type of cells and route of administration, facing several limitations including low retention in the site of the delivery or a potential immunogenicity (123). Importantly, throughout the recent years there has been an accumulating evidence that the pro-regenerative effect of SCs in the AMI treatment is caused by their paracrine activity that triggers endogenous repair mechanisms and provides immunomodulatory signaling, rather than by their direct differentiation and proliferation in the site of administration (124). Indeed, recent years of studies have brought mounting evidence on protective and pro-regenerative capability of SC-derived EVs in the treatment of AMI and other CVD-related conditions. Thus, due to the unsatisfactory results of cell-based approaches, there has been an increased focus on the alternative solutions, including those related to the utilization of EVs that not only mimic several functional properties of cells of their origin, but also are non-tumorigenic, easy to be stored and may penetrate biological barriers more effectively than the whole cells (125).

It has been widely postulated that EVs from different cell sources may potentially modulate the local microenvironment in a heart tissue toward a regeneration, exhibiting beneficial potential in CVDs treatment (**Table 1**) (132). The mechanism of EV activity is related to their transfer of bioactive cargo, mainly miRNAs, that are known to be involved in the regulation of cellular processes within a cardiac tissue (133). As an example, pro-regenerative capacity of EVs secreted by iPSC-derived cardiomyocytes was demonstrated to be mediated by the miRNA, indicating the role of miR-106a-363 cluster that represses Notch3 signaling (134). EVs isolated from human iPSCs were also shown to be enriched in several different mRNA and miRNA that may be transferred into human heart-derived cells *in vitro*, improving their cardiac and endothelial differentiation potential, as well as exhibiting cytoprotective effects (135). Similarly, murine iPSCs-EVs were shown to exhibit anti-apoptotic effect in the murine ischemia/reperfusion (I/R) model *via* the delivery of their miR-21 and miR-210 (136). Important role of miR-210 was also reported for MSCs-EVs, that were able to enhance angiogenesis *in vitro*, as well as *in vivo* in murine AMI model. The mechanism of their action was related to the inhibition of Efna3 gene expression, that is known target of miR-210, acting as an angiogenic suppressor (57). As inflammatory process is indispensably related to the cardiac failure, immunomodulatory properties of MSCs-EVs that alleviate immunological response in the site of injury are of particular focus. The role of the miRNA transfer in the immunomodulatory activity of MSC-EVs was demonstrated, pointing a role of miR-182 that inhibited toll-like receptor 4 signaling and thus promoted macrophage polarization from pro-inflammatory to anti-inflammatory phenotype in the murine I/R injury model (137). Additionally, several papers have already indicated cytoprotective and pro-angiogenic effects of MSC-EVs, including murine (138) and rat model of myocardial infarction (127). In another study, administration of EVs secreted by the murine iPSCs improved heart function *in vivo* in the infarction-reperfusion model, without any signs of teratoma, in contrary to the injection of whole cells. Additionally, the therapeutic effect of those EVs was higher when compared to the group of animals treated with iPSCs, resulting in the greater improvement in left ventricle systolic function (128). Promising results of EV use in the small animal models encouraged scientists to follow attempts to test their efficacy also in a large animal models, which are an important step toward translating basic research into clinical practice. Porcine model seems to be the most optimal for the purpose of CVDs due to the several similarities in heart size and coronary circulation to the human heart (139). One of the first studies on the porcine model of AMI have demonstrated that the intracoronary injection of conditioned medium (CM) obtained from the MSCs culture significantly increased left ventricular ejection fraction (LVEF), decreasing the size of infarct zone and reducing the oxidative stress in the residual cells (140). Few years later similar results were also presented for the CM collected from porcine EPCs (141). MSCs-EVs were also used in the nonhuman primate AMI model, demonstrating improved cardiac functions and angiogenesis following vesicle administration, pointing out an important involvement of miR-486 signaling in those processes (142).

**Table 1:** Examples of EV use in preclinical studies related to CVDs treatment.

Source of EVs	Model	Major outcomes	References
Murine ESCs	*In vivo* murine I/R model	Augmented neovascularization Enhanced cardiomyocyte survival Reduced fibrosis	(126)
Human BM-MSCs	*In vitro*	Promoted proliferation, migration, and tube formation of HUVEC	(127)
	*In vivo* rat AMI model	Promoted angiogenesis Improved hemodynamic parameters Reduced infarct size	
Murine iPSCs	*In vitro*	Enhanced angiogenic capacity, migration, and survival of cardiac endothelial cells	(128)
	*In vivo* murine I/R model	Improved LV systolic function Induced vascularization Reduced apoptosis and hypertrophy	
Human ESC-CVPCs	*In vitro*	Improved cardiomyocyte cell viability and survival Promoted cell migration and tube formation of HUVEC	(105)
	*In vivo* murine AMI model	Promoted angiogenesis Improved cardiomyocyte survival Reduced scar size	
Human CDCs	*In vivo* porcine AMI model	Decreased infarct size Preserved LV function	(129)
	*In vivo* porcine CMD model	Attenuated adverse ventricular remodelling Reduced scar Increased proliferation of cardiomyocytes in the peri-infarct zone	
Murine BM-MSCs	*In vivo* murine model of atherosclerosis	Decreased area of atherosclerotic plaques Promoted M2 macrophage polarization	(130)
Murine AT-MSCs	*In vitro*	Decreased adhesion of monocytes to AoEC	(131)
	*In vivo* murine model of atherosclerosis	Reduced atherosclerotic plaque Decreased inflammatory activation of AoEC	

AMI, acute myocardial infarction; AoEC, aortic endothelial cells; AT-MSCs, adipose derived MSCs; BM-MSCs, bone marrow MSCs; CDCs, cardiosphere-derived cells; CMD, myocardial disease; CVPCs, ESC-derived cardiovascular progenitor cells; ESCs, embryonic stem cell; HUVEC, human umbilical vein endothelial cells; MSCs, mesenchymal stem/stromal cells; iPSCs, induced pluripotent stem cells; LV, left ventricle.

Therapeutic effects of EVs were also demonstrated in the CMD model studies, dedicated for an investigation of approaches that would primarily reduce chronic inflammatory state, scar fibrosis and cardiac tissue remodelling, which are a major hallmark of chronic cardiac disfunctions that lead to adverse clinical outcome (143). Cardiac fibrosis is a consequence of differentiation of cardiac fibroblasts into myofibroblasts and their excessive ECM deposition to replace dead cardiomyocytes following an acute injury and inflammatory signaling (144). However, fibrosis-related chronic disfunction of the cardiovascular system may be also a consequence of other factors, such as aging, diabetes mellitus or other metabolic disfunctions with an inflammatory background (145). In the context of EV-based CMD therapeutic approaches, EVs from cardiosphere-derived cells were shown to prevent cardiac remodelling and improve survival in murine non-ischaemic dilated cardiomyopathy model (146), as well as in the rat model of myocarditis (147).

Atherosclerosis is also one of common CVDs that has a strong inflammatory background. It results from the plaque formation inside the large arteries that narrow the vessel lumen. Chronic inflammation plays a pivotal role in the development and progression of atherosclerosis, starting from the activation of resident endothelial cells. Subsequently, it leads to the monocyte and leukocyte recruitment into atheroma, followed by the upregulation of pro-inflammatory cytokines, production of reactive oxygen species (ROS) and matrix metalloproteinases, consequently triggering thrombotic cascade which may lead to the AMI (148). SCs-EVs display a beneficial effect in the context of atherosclerosis treatment. As an example, administration of BM-MSCs-derived EVs into high-fat diet ApoE^-/-^ mice stimulated M2 polarization of residual macrophages, which led to the decrease in the inflammation and reduction of atherosclerotic plaque area. The mechanism of EV action was possibly related to the transfer of miR-let7 family that regulated activity of downstream signaling pathways, such as NF-κB and PTEN (130). Similar immunomodulatory effect was also shown for AT-MSC-EVs, that diminished inflammatory activation of both aortic endothelial cells stimulated with tumor necrosis factor alpha (TNF-α), as well as LPS-stimulated macrophages *in vitro*, reducing atherosclerotic plaque *in vivo* in low-density lipoprotein (LDL) receptor deficient (Ldlr^-/-^) mice fed with a high-fat diet (131). In another study, EVs from UC-MSCs inhibited activation of eosinophils treated with oxidized LDL and promoted their apoptosis. This effect was even greater for EVs secreted by UC-MSCs overexpressing miR-100, with indicated role of frizzled 5 (FZD5)/Wnt/β-catenin pathway downregulation involved in this process. Decreased inflammation and atherosclerotic plaque following EVs treatment was also confirmed in this study in the murine *in vivo* model (149).

Taking together, SCs-EVs may be a promising factors for CVDs treatment, relying on their immunomodulatory and pro-regenerative activity.

#### 3.2.2 Neurological and neurodegenerative disorders

The central nervous system (CNS)- associated disorders are one of the leading causes of disability and death worldwide. Apart from malfunctions associated with either cancerous processes or acute injuries such as traumatic brain and spinal cord injury or ischemic stroke, neurodegenerative diseases are common feature among CNS pathologies, with prognosed rise in their frequency caused by the increasing life expectancy. They include the most commonly recognized malfunctions such as PD, AD, Huntington’s disease or multiple sclerosis (150).

The molecular mechanisms underlying CNS-associated disorders are still poorly understood, but several studies indicate that inflammatory processes play an essential role in their development and progression (151). Thus, further studies are required to fully delineate and develop new approaches of their effective treatment. Among them, use of SCs and their EVs occurred to be a promising strategy (152), with the latter ones being of special focus due to their ability to overcome challenges associated with crossing the BBB. Thus, during a last decade EV-based treatments of CNS-associated malfunctions have emerged as potential therapeutic candidates, with several studies reporting neuroprotective effects of EVs secreted by the SCs (**Table 2**) (160). In *in vitro* models, MSCs-derived EVs were demonstrated to reduce apoptosis, promote proliferation and stimulate secretion of pro-neurotrophic factors by neuroblastoma cell lines (161). On the other hand, EVs produced by AT-MSCs were shown to stimulate differentiation of neural progenitors, influencing miRNA and cytokine expression in the target cells (162). Comparative study demonstrated the ability of EVs derived from both MSCs and iPSCs cells to enhance the astrocyte recovery after irradiation, however vesicles obtained from MSCs exerted superior immunomodulatory effects (163). In another study, EVs secreted by iPSCs-derived neural stem cells were reported to be enriched in miRNAs and proteins known to be involved in neuroprotection, synaptogenesis and cytoprotection, possessing anti-inflammatory activity *in vitro*, as well as *in vivo* in the murine model of status epilepticus, following their intranasal administration (156). Improved recovery and angiogenesis together with reduced neuroinflammation were also reported following injection of EVs from BM-MSCs in rat models of spinal cord injury (164) and traumatic brain injury (165). Similarly, in murine model of focal cerebral ischemia MSCs-derived EVs exerted neuroprotection and neovascularisation, resulting from the regulation of the immune response in the site of injury (166). Apart from the rodent models, neuroprotective activity of human neural stem cell-derived EVs was also reported in porcine model of ischemic brain stroke, where authors presented data confirming reduced edema and intracranial haemorrhage following intravenous administration of EVs (155).

**Table 2:** Examples of EV use in preclinical studies related to the therapy of neurological and neurodegenerative disorders.

Source of EVs	Model	Major outcomes	References
Human UC-MSCs	*In vivo* rat PD model	Promoted proliferation of SH-SY5Y cells Reduced dopaminergic neuron loss and apoptosis Increased level of the striatum Relief of an asymmetric rotation defect	(153)
Murine BM-MSCs	*In vivo* murine AD model	Reduced level of amyloid plaques Decreased number of dystrophic neurites	(154)
Human NSCs	*In vivo* porcine ischemic stroke model	Decreased relative swelling of the brain Eliminated intracranial haemorrhage Improved neural tissue preservation and functional levels	(155)
Human iPSCs-derived neural stem cells	*In vitro*	Decreased release of IL-6 from macrophages	(156)
	*In vivo* murine model of epilepticus status	Enhanced hippocampal neurogenesis Reduced epileptic state Enhanced neurogenesis in hippocampus Reduction of proinflammatory cytokines in hippocampus	
Human AT-MSCs	*In vitro* HD model	Reduced accumulation of mHtt aggregates Increased activation of mitochondria Reduced apoptosis of neural stem cells	(157)
Human PMSCs	*In vitro*	Promoted maturation of oligodendrocytes	(158)
	*In vivo murine* autoimmune EAE MS model	Improved motor function Increased spinal cord myelination	
Murine BM-MSCs combined with LJM-3064 aptamer	*In vivo* murine MS model	Reduced inflammatory cell infiltration into CNS Protected CNS demyelination Increased percentage of Tregs	(159)

AD, Alzheimer’s disease; AT-MSCs, adipose derived MSCs; BM-MSCs, bone marrow MSCs; DCs, dendritic cells; CNS, central nervous system; EAE, encephalomyelitis; HD, Huntington’s disease; iPSCs, induced pluripotent stem cells; MS, multiple sclerosis; MSCs, mesenchymal stem/stromal cells; NSCs, neural stem cells; PD, Parkinson’s disease; PMSCs, placental derived MSCs; Tregs, regulatory T cells; UC-MSCs, umbilical cord Wharton’s jelly MSCs.

Protective role of EVs was also shown in the several models of neurodegenerative diseases (167). As an example, administration of neuroblastoma-derived EVs lowered the level of amyloid-β peptide (Aβ) that is known to be elevated in AD (168). Similarly, intracerebral injection of MSC-derived EVs in the murine model of AD reduced the level of amyloid plaques, mediated by the transfer of neprilysin protein known as a endopeptidase able to degrade Aβ (154). EVs were also employed as a drug delivery system in murine model of PD, by their loading with antioxidative catalase followed by the EV intranasal delivery, exerting neuroprotective and anti-inflammatory effects *in vitro* and *in vivo* (91). Moreover, in rat model of PD animals treated intranasally with EVs secreted by human exfoliated deciduous teeth SCs exhibited improved gait parameters (169). In another study, MSCs-EVs were shown to cross BBB in rat PD model and lower dopaminergic neuron loss in substantia nigra, concomitantly with an increased level of striatum (153). Protective role of SCs-EVs was also reported for the treatment of multiple sclerosis (MS), as neurodegenerative disease of CNS with the inflammatory background related to the BBB dysfunction and chronic activation of lymphocytes against oligodendrocyte proteins, that leads to the demyelination and synaptopathy (170). As an example, EVs from placental MSCs improved motor function and spinal cord myelination in autoimmune encephalomyelitis murine MS model (158). In another approach, MSCs-EVs were combined with LJM-3064 aptamer with previously demonstrated ability to induce remyelination. Such engineered hybrid particles exhibited anti-inflammatory activity and protected against CNS demyelination in murine MS model *in vivo* (159). Altogether, there has been accumulating evidence on the role of EVs in the treatment of different types of CNS-associated disorders.

#### 3.2.3 Kidney injury

Proper functioning of kidneys is essential for the effective control of body fluids osmolarity, pH and removal of toxic metabolites. Thus, kidney injuries are life-threatening conditions resulting in the dysregulation of homeostasis (171). One of the most severe kidney disorders is acute kidney injury (AKI) that accompanied by the systemic inflammation leads to the rapid damage of organ structure followed by a loss of renal function, with the need of patient hospitalisation, high mortality rate and high risk of the development of chronic kidney dysfunction (172). Thus, the development of effective therapeutic approaches for the AKI treatment is an important challenge of the modern medicine. EVs play an important role not only as prognostic factors and biomarkers of renal disfunction, but have also been demonstrated as potential new-generation tools for the therapy of AKI (**Table 3**) (180). Importantly, an inflammatory response accompanying AKI has been widely reported to be significantly ameliorated by EVs from MSCs *via* their immunomodulatory stimuli. Meta-analysis study collecting the data from 31 preclinical studies on rodents have confirmed the therapeutic efficacy of MSC-EVs in AKI treatment (181). As an example, in the rat renal ischemia-reperfusion injury model, BM-MSCs-derived EVs inhibited apoptosis and stimulated tubular epithelial cell proliferation (182). In other study, EVs derived from native, but not from interferon gamma (IFN-γ)- stimulated UC-MSCs were able to alleviate the effect of hypoxia-induced AKI in the rat model (183). As nephrotoxicity is an important issue in oncological patients, being caused by the widely-used chemotherapeutic agents such as cisplatin, there is a need for the new therapeutic strategies that would reduce severe side effects related to the chemotherapy and improve clinical outcomes of patients. In the studies where rat cisplatin-induced AKI model was used, EVs secreted by UC-MSCs (184) and AT-MSCs (185) were able to exhibit cytoprotective activity, reducing cell death and inflammatory response. Additionally, in the murine model of cisplatin-induced AKI, EVs secreted by BM-MSCs improved renal function, but the effect was dependent on the route of EV administration, with multiple injections being beneficial over the single dose (186). The pro-regenerative activity in the context of renal function was also demonstrated for EVs from different types of cells. For instance, EVs from amniotic epithelial cells were shown to reduce nephrotoxicity in the murine model of cisplatin-induced AKI (187). ESC-EVs were also demonstrated to exhibit pro-regenerative effect in the murine model of ischemia-reperfusion AKI, by stimulating angiogenesis and proliferation of renal epithelial cells, as well as reducing renal fibrosis. These observations correlated with the activation of the resident Sox9+ cells that are known to be involved in the processes of formation and regeneration of renal tubular epithelium (104).

**Table 3:** Examples of EV use in preclinical studies related to the treatment of kidney diseases.

Source of EVs	Model	Major outcomes	References
K-MSCs	*In vivo* AKI murine model	Promoted angiogenesis Decreased cell apoptosis	(173)
Murine BM-MSCs	*In vitro*	Reversed changes in the morphology and expression of E-cadherin and α-SMA in HK2 cells	(174)
	*In vivo* murine CKD model	Protection against unilateral ureteral obstruction Enhancement of the expression of α-SMA and E-cadherin in kidney Reduced tubular damage	
	*In vitro*	Suppressed ER stress Protection of cells against damage and apoptosis Promoted proliferation of renal tubular epithelium	(175)
	*In vivo* murine kidney I/R model	Suppressed ER stress Protection against renal I/R injury	
	*In vitro*	Attenuated morphological changes and restored EMT in HK2 cells	(176)
	*In vivo* murine UUO model	Ameliorated renal function Decreased interstitial lymphocyte infiltration	
Murine AT-MSCs	*In vivo* murine AKI model	Promoted functional kidney recovery Decreased apoptosis of tubular epithelial cells	(177)
	*In vivo* rat CKD model	Reduced pathological changes and renal fibrosis Protection of kidneys against inflammation, mitochondrial dysfunction, and apoptosis	(178)
Rat BM-MSCs	*In vitro*	Prevented SMAD2/3 and ERK1/2 phosphorylation in HK2 cells	(179)
	*In vivo* rat CKD model	Inhibition of renal fibrosis Ameliorated renal function and morphology	

AKI, acute kidney injury; AT-MSCs, adipose derived MSCs; BM-MSCs, bone marrow MSCs; CKD, chronic kidney disease; EMT, epithelial-mesenchymal transition; ER, endoplasmic reticulum; HK2, human kidney 2 cells; I/R, ischaemia-reperfusion; K-MSCs, kidney-derived MSCs; UUO, unilateral ureteral obstruction; αSMA, alpha smooth muscle actin.

Apart from AKI, EVs were shown to exhibit immunoregulatory, cytoprotective and pro-regenerative activity in a treatment of chronic kidney disease (CKD) that leads to the progressive nephropathy. One of the important causes of CKD is renal hypoxia and persistent inflammation, which lead to the kidney fibrosis (188). Due to the complexity of CKD pathogenesis, current pharmacological treatments are unsatisfactory (189). The therapeutic effect of EVs in CKD treatment was demonstrated in meta-analysis covering the results from 35 studies, that mostly based on the unilateral ureteral obstruction (UUO) model of this disease (190). Protective, anti-inflammatory and anti-fibrotic role of MSC-EVs in the chronic renal dysfunction was observed both *in vitro* and *in vivo* (176), with an indication on an important role of EV-based miRNA transfer involved in those processes (**Table 3**) (179).

#### 3.2.4 Liver disfunctions

Liver disfunctions, including acute injuries and chronic diseases, are considered as a significant burden experienced by many individuals, that may consequently lead to the life-threatening conditions such as end-stage cirrhosis, fibrosis or liver malignancies. Still, one of the standard therapeutic approaches is a liver transplantation. However, due to the limited availability of donors, mortality from liver-related malfunctions continues to be a critical issue, that raises the urgent need for an effective, alternative therapies for the liver replacement (191).

Liver-related diseases may be caused by alcohol, drugs, metabolic diseases or viral hepatitis. In terms of the treatment of liver disfunctions, the major goal is to inhibit fibrosis related to the chronic liver disease, that causes hepatic dysfunction, activation of hepatic stellate cells, excessive deposition of ECM and immunological response to the local inflammation (192). Thus, anti-fibrotic and anti-inflammatory therapeutic strategies including treatment with EVs, are of current interest. Indeed, several experimental approaches have demonstrated the effectiveness of different types of SC-derived EVs in ameliorating liver disfunctions (**Table 4**). As an example, iPSC-derived EVs were shown to supress fibrosis in two murine models of liver injury, caused by either treatment with CCl_4_ or by bile duct ligation (193). EVs secreted by MSCs differentiated from ESCs were also reported to alleviate thioacetamide-induced chronic liver injury, reducing cirrhosis and pro-fibrotic production of collagen I and α-smooth muscle actin (αSMA), with simultaneous decrease in the pro-apoptotic and pro-inflammatory factors (198). Similar antifibrotic effect was also demonstrated in CCl_4_-induced liver fibrosis for EVs isolated from UC-MSCs and the mechanism of their action was related to the inhibition of epithelial-to-mesenchymal transition (EMT) of hepatic cells (194). In another study, UC-MSCs-derived EVs were also demonstrated to ameliorate acute liver injury due to the antioxidative and antiapoptotic effect (199). It was also shown that hepatocyte-derived EVs are able to alleviate inflammatory response and pro-fibrotic activation of hepatic stellate cells, as well enhance proliferation of hepatocytes, both *in vitro* and *in vivo* in the murine model of CCl_4_ injury (195).

**Table 4:** Examples of EV use in preclinical studies related to the treatment of liver dysfunctions.

Source of EVs	Model	Major outcomes	References
Human iPSCs	*In vitro*	Modulation of the profibrogenic transcriptome profile in activated HSCs	(193)
	*In vivo* murine model of ameliorating liver disfunctions	Reduced development of fibrosis	
Human UC-MSCs	*In vivo* murine CCl4-induced liver fibrosis model	Reduced development of fibrosis Reduced expression of collagen I and III Inactivation of TGF-b1/Smad signaling pathway	(194)
Mouse hepatocytes	*In vivo* murine hepatic fibrogenesis model	Reduced inflammation Reduced development of fibrosis Suppressed monocyte/macrophage infiltration	(195)
LX-2	*In vitro*	Decreased proliferation and invasion of HCC	(196)
	*In vivo* murine model of HCC	Reduced tumor size Increased apoptosis of HCC	
Human AT-MSCs	*In vitro*	Increased chemosensitivity of HCC cells	(197)
	*In vivo* murine model of HCC	Increased sensitiveness of HCC to chemotherapeutic agents	

AT-MSCs, adipose derived MSCs; HCC, hepatocellular carcinoma; HSCs, hepatic stellate cells; iPSCs, induced pluripotent stem cells; UC-MSCs, umbilical cord Wharton’s jelly MSCs.

#### 3.2.5 Respiratory system diseases

Involvement of EVs in the respiratory system is also well documented, with their role not only as potential biomarkers, but also as therapeutic agents, regulating the immune cell functions during airway inflammatory diseases (**Table 5**) (207). Particularly, MSCs-EVs hold a great promise as factors mimicking beneficial immunomodulatory properties of their parental cells, thus augmenting inflammatory response typically associated with the respiratory system malfunction and tissue damage (208). Additionally, possibility to administer EVs *via* inhalation facilitates their entry into pulmonary system and targeted delivery of their cargo into the site of interest.

**Table 5:** Examples of EV use in preclinical studies related to the treatment of the respiratory system diseases.

Source of EVs	Model	Major outcomes	References
Human UC-MSCs	*In vivo* murine model of asthma	Reduced inflammatory response and airway remodelling Prevented lung remodelling Reduced inflammatory cell infiltration Decreased level of pro-inflammatory cytokines Inhibited TGF-β1-Smad2/3 signaling pathway	(200)
	*In vivo* murine model of lung ischemia-reperfusion injury	Attenuated inflammation and edema Attenuated activation of iNKT cells and macrophages Decreased level of pro-inflammatory cytokines Inhibition of macrophage and iNKT cells activation	(201)
Porcine BM-MSCs	*In vitro*	Inhibition of virus replication in lung epithelial cells Inhibition of virus-induced apoptosis replication in lymphatic endothelial cells	(202)
	*In vivo* porcine model of influenza-induced ALI	Inhibition of virus replication in lung epithelial cells Reduced lung injury Attenuated level of pro-inflammatory cytokines	
Human BM-MSCs	*In vivo* murine model of lung injury	Decreased lung vascular endothelial permeability	(203)
	*Ex vivo* human model of pneumonia	Improved alveolar fluid clearance in lungs Reduced level of bacteria	(204)
Human Amnion Epithelial Cells	*In vivo* murine model of idiopathic pulmonary fibrosis	Prevention against lung injury	(205)
Human iPSC-MSCs	*In vivo* murine model of asthma	Ameliorated allergic airway inflammation Alleviation of airway hyperresponsiveness Decrease of inflammatory cell infiltration	(206)

ALI, acute lung injury; BM-MSCs, bone marrow MSCs; iNKT, invariant natural killer T cells; iPSCs, induced pluripotent stem cells; MSCs, mesenchymal stem/stromal cells; UC-MSCs, umbilical cord Wharton’s jelly MSCs.

Among the variety of lung diseases, one of the most severe conditions are related to the acute lung injury (ALI) and acute respiratory distress syndrome (ARDS) that carry high morbidity and mortality rates, resulting from the rapid respiratory failure (209). In porcine model of influenza-induced ALI, intratracheal administration of porcine MSC-EVs resulted in diminishing lung injury, inhibition of virus replication in lung epithelial cells *in vitro* and *in vivo* and reduction of inflammation within the lung tissue (202). MSCs-EVs were also shown to alleviate alveolar inflammation and pulmonary edema in *E. coli* endotoxin-induced ALI (90). Similarly, in *ex vivo* perfused human model of *E. coli*-driven pneumonia, MSCs-EVs increased alveolar fluid clearance and antimicrobial activity of macrophages. This effect was even enhanced by the pre-treatment of parental MSCs with Toll-like receptor 3 agonist (204). In the murine model of lung injury EVs secreted by BM-MSCs decreased the lung vascular endothelial permeability caused by the of haemorrhagic shock, with possible involvement of the mechanism related to the reduction of cytoskeletal RhoA signaling activity (203). In addition, anti-inflammatory, protective and/or regenerative properties of MSC-EVs have also been observed in rodent models of pulmonary hypertension (210), radiation-induced injury (211), bronchopulmonary dysplasia (212) and idiopathic pulmonary fibrosis (213). In the last one the regenerative effect has been also demonstrated for EVs secreted by iPSCs (214). Additionally, in the murine model of lung ischemia-reperfusion injury, administration EVs derived from MSCs attenuated inflammation and edema (201). Similar outcome was also reported in the rat model, indicating an influence of EVs on the expression of genes regulating inflammation and oxidative stress (215).

Beneficial effect was also shown for MSCs-EVs in rodent models of asthma as one of the common manifestations of immune system overactivation. As an example, it was demonstrated that vesicles secreted by UC-MSCs were able to reduce inflammatory response and airway remodelling. Importantly, this effect was boosted for animals that received EVs from hypoxia-stimulated cells (200). Similarly, MSCs-EVs were shown to inhibit group 2 innate lymphoid cells (ILC2s) that are known to be involved in the pathogenesis of airway allergy. Additionally, those EVs were able to reduce the level of pro-inflammatory cytokines and mucus production in the murine model of asthma, with the suggested role of miR-146a transfer involved in this effect (206).

#### 3.2.6 Digestive system dysfunctions

Anti-inflammatory and immunomodulatory properties of EVs make them a promising option for the treatment of diseases associated with the digestive system that are typically related to the multimodal gut inflammation (216). Indeed, several attempts were performed in this field so far (**Table 6**). As an example, in the murine *in vivo* model of ulcerative colitis induced by the dextran sulphate sodium treatment, EVs from BM-MSCs ameliorated disease symptoms, including colon mucosa damage, by stimulating polarization of macrophages into anti-inflammatory M2 phenotype through the modulation of JAK/STAT signaling pathway (217). In another study, a suppressive influence of BM-EVs on macrophage activity was also demonstrated in murine model of inflammatory bowel disease (IBD), resulting in an improved gut functions and decreased mucosal inflammation (222). From another point of view, EVs from M2 macrophages were also reported to attenuate colitis in mice and their mode of action was related to the stimulation of Tregs *via* CCL1 chemokine (218).

**Table 6:** Examples of EV use in preclinical studies related to the treatment of the digestive system disorders.

Source of EVs	Model	Major outcomes	References
Murine BM-MSCs	*In vivo* murine model of ulcerative colitis	Attenuated colon mucosa damage Promoted polarization of M1 macrophages to the M2 state Suppressed inflammatory response	(217)
Murine M2 macrophages	*In vivo* murine model of colitis	Attenuated colitis Alleviated colon damage Increased percentage of Tregs Decreased level of pro-inflammatory cytokines	(218)
Grapefruit pulp	*In vivo* murine model of DSS-induced colitis	Enhanced anti-inflammatory capacity of intestinal macrophages Maintained intestinal macrophage homeostasis Decreased level of pro-inflammatory cytokines	(219)
Murine blood serum	*In vivo* murine DSS-induced colitis	Decreased permeability in colon tissues	(220)
Murine Tregs	*In vitro*	Promoted proliferation and inhibited apoptosis of YAMC cells	(221)
	*In vivo* murine model of DSS-induced colitis	Alleviated IBD	
Human BM-MSCs	*In vivo* murine model of IBD	Suppressed inflammatory response Reduced development of fibrosis Promoted M2 polarization of macrophages Decreased permeability of colon tissue	(222)

BM-MSCs, bone marrow MSCs; DSS, dextran sulfate sodium; IBD, inflammatory bowel disease; Tregs, regulatory T cells; YAMC, conditionally immortalized mouse colon epithelial cell line.

#### 3.2.7 Skin damage

Skin as the largest organ in the body plays an important role in the maintenance of homeostasis and provides a protective barrier against external hazardous factors, thus, is constantly exposed to potential severe injuries, including thermal and chemical burns, chronic wounds or persistent microbial infections, that may lead to the fatal trauma (223). SCs-EVs were used for the treatment of inflammatory skin diseases (**Table 7**). As an example, EVs from AT-MSCs diminished symptoms of atopic dermatitis in the murine *in vivo* model of this disease, induced by the dust mite treatment of animals. Following administration of EVs the number of eosinophils and serum IgE decreased, together with the reduction of pro-inflammatory cytokines levels in the skin lesions (230). Similarly, in the *in vivo* model of oxazolone-induced dermatitis, AT-MSCs-EVs reduced inflammation, as well as improved ceramide production and epidermal barrier, preventing skin water loss (231). In another study, EVs from UC-MSCs reduced excessive proliferation of epidermis cells, decreased expression of interleukin IL-17 and IL-23, as well as inhibited activation of DCs is the murine model of psoriasis (227).

**Table 7:** Examples of EV use in preclinical studies related to the treatment of skin dysfunctions.

Source of EVs	Model	Major outcomes	References
Human iPSCs	*In vitro* model of skin aging	Increased proliferation and migration of skin fibroblasts Decline in UVB-stimulated photoaging Decreased level of matrix-degrading enzymes	(224)
Human iPSCs-derived MSCs	*In vivo* rat skin wound healing model	Enhanced angiogenesis Increased proliferation of the skin Improved reepithelialisation	(225)
Human UC-MSCs	*In vivo* murine full-thickness skin wound model	Promoted proliferation and migrative of endothelial cells and skin fibroblast Improved re-epithelialisation Reduced level of proliferation suppressor genes	(226)
	*In vivo* murine model of psoriasis	Reduction of excessive epidermis proliferation Decreased level of pro-inflammatory cytokines	(227)
Human BM-MSCs	*In vitro*	Promoted viability of fibroblast, keratinocyte, and endothelial cells Induced endothelial cell migration	(228)
	*In vivo* murine model of diabetic skin healing	Accelerated wound closure Increased epithelial thickness	
	*In vivo* rat diabetic wound healing model	Increased macrophage M2 polarization Enhanced angiogenesis and healing Suppressed level of pro-inflammatory factors	(229)
Human AT-MSCs	*In vivo* murine model of atopic dermatitis	Decreased number of eosinophils and serum IgE Decreased level of pro-inflammatory cytokines Reduced inflammation	(230)

AT-MSCs, adipose derived MSCs; BM-MSCs, bone marrow MSCs; iPSCs, induced pluripotent stem cells; MSCs, mesenchymal stem/stromal cells; UC-MSCs, umbilical cord Wharton’s jelly MSCs.

Recent studies have also shown beneficial effect of EVs in skin regeneration (232). For instance, subcutaneously injected EVs isolated from iPSCs-derived MSCs enhanced angiogenesis and re-epithelialisation, leading to the wound closure. Additionally, they also stimulated proliferation of skin fibroblasts and ECM production (225). In another study of murine full-thickness skin wound model, EVs from UC-MSCs promoted proliferation and migrative capacity of both endothelial cells and skin fibroblast, as well as improved angiogenesis *in vitro*, with improved re-epithelialisation demonstrated *in vivo* (226). Similarly, in the context of chronic wound treatment, UC-MSCs-derived EVs applied in the hydrogel formulation onto the wound accelerated skin healing and regeneration in the diabetic rat model (233). Interestingly, EVs from AT, but not from BM were able to enhance skin healing in murine model of diabetic murine model. These differences corresponded to the differential cargo in both types of EVs, with predominant role of BM-MSCs-EVs and AT-MSCs in promotion of skin cells proliferation and angiogenesis, respectively (228). On the other hand, in another study there was no significant difference in the pro-regenerative potential of MSCs from both BM and AT in such model, which may indicate the variance in the mechanism of action between cells and their secretory vesicles (234). An importance of immunomodulatory signaling mediated by MSCs-EVs was also demonstrated for the skin damage treatment. As an example, EVs from melatonin-preconditioned BM-MSCs triggered macrophage M2 polarization, resulting in the decrease of pro-inflammatory cytokines and increase in the expression of anti-inflammatory IL-10, enhancing angiogenesis and healing in rat diabetic wound model (229).

EVs derived from iPSCs may be also used for the purpose of skin regeneration. Importantly, due to the higher “stemness” potential of iPSCs when compared to MSCs, scientist attempt to utilize these properties in the context of antiaging skin treatment. As an example, dermal fibroblasts treated with hiPSCs-EVs possessed higher proliferative capability and thus lowered senescence. Additionally, UVB-stimulated photoaging process in those cells was also decreased following hiPSCs-EVs treatment (224). Similar results were obtained by another group, which demonstrated that “cell-engineered nanovesicles” obtained by the serial membrane extrusion of human iPSCs augmented senescent alterations in skin fibroblasts (235). Nevertheless, EVs from MSCs were also used in several studies related to the protection against skin aging. In one of studies, AT-MSCs-derived EVs attenuated UVB-triggered photoaging both *in vitro*, as well as in the murine *in vivo* model, and their mechanism of action was related to the inhibition of inflammatory-induced macrophage differentiation and ROS production, resulting in lower wrinkle scoring (236). Interestingly, direct comparison study have revealed higher antiaging effect of EVs derived from hiPSCs than MSCs (110).

#### 3.2.8 Pain

Fighting the chronic pain that accompanies several inflammatory-related diseases is still a challenging aspect of medicine. There are several attempts reporting the possible usage of EVs in the pain treatment (**Table 8**) (242). In one of the studies, UC-MSCs-EVs were used as a therapeutic agents in the rat model of neuropathic pain caused by the nerve injury. Intrathecal administration of EVs resulted in the reduced symptoms of pain and lower hind paw hypersensitivity, decreasing the expression of pro-inflammatory factors in dorsal root ganglion in the site of injury (237). In another report, intra-articular administration of secretome obtained from BM-MSCs stimulated with TNF-α and IFN-γ and ameliorated pain in the murine model of osteoarthritis (238). Moreover, EVs secreted by iPSCs-derived MSCs decreased tendinopathy-related pain symptoms in rat model *in vivo*, alleviating inflammation and enhancing proliferation of tenocytes (239). Not only EVs from SCs, but also immune cells may have the ability to reduce inflammation-related pain symptoms. For example, in the murine inflammatory pain model EVs from M2 macrophages were able to transfer miR-23a to microglia, increasing threshold of mechanical allodynia and thermal hyperalgesia *via* regulation of NF-E2-related factor 2 (NRF2) (241). Altogether these reports indicate that EVs may serve as a potential factors for the anti-pain treatment approaches.

**Table 8:** Examples of EV use in preclinical studies related to the pain treatment.

Source of EVs	Model	Major outcomes	References
Human UC-MSCs	*In vivo* rat model of neuropathic pain	Reduced pain symptoms Decreased the expression of pro-inflammatory factors	(237)
Human BM-MSCs	*In vivo* mouse model of osteoarthritis	Ameliorated pain Protective effect on cartilage damage	(238)
Human iPSCs-derived MSCs	*In vivo* rat model of tendinopathy-related pain	Ameliorated pain Enhanced proliferation of tenocytes Down-regulation of the gene expression-related to inflammation	(239)
Mouse NSCs	*In vivo* rat model spinal cord injury	Reduced neuronal apoptosis Decreased microglial activation Attenuated neuroinflammation	(240)
Macrophages	*In vivo* model of murine inflammatory pain	Alleviated inflammatory pain	(241)

BM-MSCs, bone marrow MSCs; iPSCs, induced pluripotent stem cells; MSCs, mesenchymal stem/stromal cells; NSCs, neural stem cells; UC-MSCs, umbilical cord Wharton’s jelly MSCs.

#### 3.2.9 COVID-19

Coronavirus infectious disease 2019 (COVID-19) caused by the severe acute respiratory syndrome coronavirus type 2 (SARS-CoV-2), was first time reported in Wuhan, China in a late 2021 and has rapidly spread over the world, emerging as a global pandemic issue. Till August 2022, COVID-19 affected more than half billion of people worldwide, causing death of more than 6 million (243). SARS-CoV-2 infects host cells by interaction of its spike protein with angiotensin converting enzyme 2 (ACE2) receptor, present on several types of epithelial and endothelial cells (244). Main clinical manifestations of this disease are related to the respiratory system, including strong cough, hypoxia, pneumonia and ARDS. However, it may also manifest by multiorgan disfunction, including cardiovascular, nervous or gastrointestinal system. COVID-19 is typically accompanied by mild to moderate flu-like inflammatory symptoms such as fever, muscle ache and general weakness, but in many individuals may lead to the acute cytokine storm, sepsis and in a consequence death (245). Long-term post-COVID complications were also widely reported, with multiple health issues that may last for several months from the moment of infection (246). COVID-19 outbreak has not only caused a death of many people, but also dramatically affected international economy, impacted global healthcare and negatively influenced a social life (247). Thus, increasing number of cases has raised a global pressure to find effective ways of COVID-19 prevention and effective treatment. Despite the rapid development of emergency vaccination, still its accessibility is not uniform, with accompanied hesitancy of the part of the society against the common vaccination. Additionally, there’s a lack of specific and highly effective treatment against COVID. One of the crucial issues is to inhibit uncontrolled hyperactivation of immune system that leads to the cytokine storm and consequently to the multiorgan damage (248).

It was shown that EVs may be considered not only as biomarkers of COVID-19 outcome (249), but also as immunomodulatory agents that may ameliorate inflammatory complications and improve the clinical outcome of patients (**Table 9**) (254). In this respect, MSCs-EVs are predominantly tested as cell-free alternatives mimicking immunosuppressive properties of their cells of their origin. As an example, the potential of EVs from UC-MSCs to decrease the release of pro-inflammatory cytokines was demonstrated *in vitro* on human lung adenocarcinoma epithelial cells stimulated with SARS-CoV-2 peptides (250). Another study has demonstrated safety and efficacy of intravenous administration of BM-MSCs-derived EVs to 24 COVID-19-positive patients with moderate or acute ARDS. Additionally, following EV treatment an improved oxygenation ratio and decreased inflammatory status was also reported, which opened a possibility for the further studies, including clinical trials on the higher number of patients (251). Interestingly, elevated number of EVs possessing ACE2 receptor were found in the plasma of COVID-19 patients and were shown to inhibit binding of viruses and their spike protein to HEK cells *in vitro*, as well as to ameliorate severity of this disease in the rodent model (252). There are also attempts to use EVs as vaccines against SARS-CoV-2 infection. As an example, EVs derived from *Salmonella typhimurium* decorated by spike receptor-binding domain were used as immunization factors in syrian hamster COVID-19 model, exerting the effective production on neutralizing antibodies against few variants of SARS-CoV-2 (253). Altogether, use of EVs as immunoregulatory factors may open a new perspectives of COVID-19 treatment and prevention.

**Table 9:** Examples of EV use in preclinical studies related to the COVID-19 treatment.

Source of EVs	Model	Major outcomes	References
Human UC-MSCs	*In vitro*	Reduced SARS-CoV2-induced inflammatory cytokines Decreased level of NF-κB-p65	(250)
Human BM-MSCs	*In vivo* SARS-CoV2 positive patent	Improved oxygenation ratio Decreased inflammatory status	(251)
Human HEK	*In vitro*	Inhibited binding of viruses to HEK cells	(252)
	*In vivo* murine SARS-CoV2 model	Ameliorated the symptoms of the disease	
*Salmonella typhimurium*	*In vivo* Syrian hamster SARS-CoV-2 model	Production on neutralizing antibodies Decreased size of inflammatory focal patches	(253)

BM-MSCs, bone marrow MSCs; HEK, human embryonic kidney cells; MSCs, mesenchymal stem/stromal cells; SARS-CoV-2, severe acute respiratory syndrome coronavirus 2; UC-MSCs, umbilical cord Wharton’s jelly MSCs.

#### 3.2.10 Osteoarthritis

Osteoarthritis (OA) is a type of chronic degenerative disease of an articular cartilage. Consequently, it leads to the progressive inflammation, pain and joint dysfunction, predominantly in the knees, but also hips and fingers. It has been indicated as one of the ten most disabling disorders in the developed countries, with about 10% of men and close to 20% of women aged over 60 years to have symptomatic OA. Apart from age, major risk factors associated with OA are joint injuries and obesity (255). Currently available therapeutic approaches are limited and concentrate mainly either on temporal, pharmacological pain relief and reduction of inflammation, or on the invasive surgical interventions and joint replacement (256).

SCs-EVs were proven to support OA treatment, with the special regard to those secreted by MSCs (**Table 10**). As an example, EVs from BM-MSCs were reported to increase the expression of type II collagen and aggrecan, with reduction of metalloproteinase 13 and iNOS, in OA-like chondrocytes *in vitro*. Additionally, they exhibited anti-inflammatory and cytoprotective effect *in vivo*, decreasing cartilage and bone degeneration in the knee joint in collagenase-induced murine OA model (257). In another study, BM-MSCs-EVs reduced expression of pro-inflammatory cyclooxygenase 2 (COX2) and NFκB signaling, with simultaneous enhancement of the proteoglycan and type II collagen level in TNF-α-stimulated chondrocytes derived from OA patients (258). Similarly, EVs isolated from AT-MSCs exhibited chondroprotective effect on IL-1β-stimulated OA chondrocytes *in vitro*, diminishing secretion of pro-inflammatory factors (TNF-α, IL-6, prostaglandin E2, nitric oxide, COX2) and increasing level of IL-10 and type II collagen (259). Furthermore, UC-MSCs-EVs had immunomodulatory effect in OA model *in vitro* and *in vivo*, promoting M2 macrophage polarization and secretion of anti-inflammatory IL-10, as well as inhibiting cartilage degradation. The mechanism of their action was related to miRNA cargo known to regulate PI3K pathway in targeted cells (260). Altogether, these data demonstrate the chondroprotective and immunomodulatory activity of EVs in the context of potential OA treatment.

**Table 10:** Examples of EV use in preclinical studies related to the treatment of OA.

Source of EVs	Model	Major outcomes	References
Murine BM-MSCs	*In vitro* model of OA	Restored homeostasis in OA-like chondrocytes Decreased apoptosis of chondrocytes Decreased expression of pro-inflammatory factors	(257)
	*In vivo* murine model of OA	Reduced degradation of cartilage and bone	
Human BM-MSCs	*In vitro* model of OA	Decreased level of pro-inflammatory cytokines Decreased expression of NF-κB-p65 Promoted production proteoglycan by chondrocytes Enhanced proliferation of chondrocytes	(258)
Human AT-MSCs	*In vitro* model of OA	Reduced production of inflammatory mediators Decreased expression of iNOS	(259)
Human UC-MSCs	*In vitro*	Increase of macrophage M2 polarization	(260)
	*In vivo* rat model of OA	Inhibited cartilage degradation	

AT-MSCs, adipose derived MSCs; BM-MSCs, bone marrow MSCs; iNOS, inducible nitric oxide synthase OA, osteoarthritis; UC-MSCs, umbilical cord Wharton’s jelly MSCs.

#### 3.2.11 Cancer

Immunoregulatory capability of EVs makes them an attractive option for the treatment of cancer, as one of the leading causes of death worldwide. Oncological immunotherapy is one of the rapidly developing treatments, targeted to stimulate immune system toward anti-cancer defence, that includes checkpoint blockade therapies, use of chimeric antigen receptor (CAR) T-cells and cancer vaccines. Currently, there are attempts to use preparations containing EVs as anti-cancer vaccines (**Table 11**) (264). This strategy relies on the use of EVs secreted by the cancer cells or by APCs, with the special focus on DCs. The latter ones were shown to contain functional MHC class I and II antigens, as well as co-stimulatory molecules capable to activate the anti-tumor response of cytotoxic T cells (265). Moreover, utilization of autologous tumor-derived EVs harbouring cancer-specific antigens as nanovaccines opens new possibilities of the development of personalized anti-cancer treatment. However, due to the low immunogenicity of autologous EVs from cancer cells, there are attempts to combine them with other factors that would enhance anti-tumor response of immune system. As an example, researchers created hybrid nanoparticles by combining EVs of tumor and *E. coli* origin, that were able to stimulate maturation of DCs and trigger strong anti-tumor immune response in colon, melanoma and breast cancer murine models (262). In another study, cell membrane vesicles from melanoma cells were combined with CpG oligonucleotides, TLR-9 agonist and DCs-targeting aptamer, enabling specific activation of immune system against cancer, together with a long-term immune memory effect (263).

**Table 11:** Examples of EV use in preclinical studies related to the treatment of cancer.

Source of EVs	Model	Major outcomes	References
Human BM-MSCs	*In vitro*	Inhibited proliferation and viability of HepG2, Kaposi, and Skov-3 cell lines	(261)
	*In vivo* murine cancer model	Inhibition of tumor growth	
*Escherichia coli* combined with tumour cells	*In vivo* murine cancer model	Stimulated maturation of DCs Regression of tumor	(262)
Murine melanoma cells combined with CpG oligos, TLR-9 agonist, and DCs-targeting aptamer	*In vivo* murine melanoma model	Stimulated maturation of DCs Stimulated specific activation of immune system against cancer	(263)

BM-MSCs, bone marrow MSCs; DCs, dendritic cells; HepG2, human liver cancer cell line; TLR-9, Toll-like receptor 9.

Additionally, SCs-EVs were also shown to exhibit anti-cancer activity. In particular, EVs isolated from BM-MSCs inhibited proliferation of HepG2 hepatoma, Kaposi’s sarcoma, and ovarian tumor cell lines, inducing cancer cell death *in vitro*, as well as exhibiting anti-tumor activity following subcutaneous injection of EVs in the *in vivo* experiments (261). Similar results were also demonstrated for UC-MSCs-derived EVs in the model of bladder tumor (266). Thus, EV-based approaches may be a novel, promising strategy for the anti-cancer therapy.

### 3.3 Challenges and perspectives

Rapidly developing knowledge on EV biology and their functions result in the growing number of attempts to use EVs as new-generation tools in the regenerative medicine, as well as in several other biomedical fields. One on them is the attempt to use EVs as biological nanoparticles for the transport and targeted delivery of drugs and other biologically active particles, which relies on an intrinsic activity of EVs as mediators of cell-to-cell communication (267). As an example, in one study curcumin-loaded EVs were able to reduce pro-inflammatory signalling in macrophages *in vitro* more effectively, when compared to the curcumin itself, which demonstrates that EV-based strategy enhances bioavailability of this low-soluble compound. Additionally, survival rate of animals in the LPS-induced sepsis model was also significantly higher for EV-curcumin group, comparing to animals treated only with curcumin (268). Similarly, EVs from immature dendritic cells were also used to deliver anti-tumor agent- doxorubicin that was loaded to them *via* the electroporation. Such EVs were then demonstrated to specifically target tumor cells, inhibiting their growth both *in vitro* and *in vivo* (269). Interestingly, there are indications that EVs may be taken up by acceptor cells more effectively when compared to the liposomes, with the simultaneous high efficiency of EV “loading” with particular bioactive molecules (270). Additionally, due to their endogenous origin, EVs are envisioned as less immunogenic and cytotoxic when compared to the synthetic nanoparticles (271). Importantly, EVs have also been shown to be able to deliver siRNA to the murine brain *in vivo*, which opens new possibilities for the development of new, drug-carrying particles capable to cross BBB, which so far is an important factor limiting the effectiveness of the neurological diseases therapy (272).

EVs are promising therapeutic options that have additional potential to be engineered, both on the level of their parental cells and after their secretion. First approach includes cell preconditioning or genetic engineering, whereas second one bases e.g. on loading of EVs with particular therapeutic compounds. Such modification of “native” EVs may help to develop approaches to either overcome limitations related to EV use or to boost their therapeutic efficacy, targeted delivery or stability, which widens further possibilities of EV utilisation in the future biomedical applications (273).

#### 3.3.1 Pitfalls and limitations of EV utilisation

Despite significant progress in the field, there are still several limitations of broader use of EVs in biomedical sciences. Translation of the basic science into the clinics encounters critical challenges and obtained EV preparations have to fulfil several stringent, but still not fully defined criteria, that include variety of quantitative and qualitative properties. Importantly, constantly increasing knowledge on EV biology raises new questions and doubts on their identity, optimal methods of isolation, as well as methodological barriers of their characterization (274). So far, several key aspects have been recognized as potential hindrances of EV utilization in pre-clinical and clinical studies.

One of the pitfalls is to obtain a pure EV fraction without accompanying non-vesicular entities such as protein complexes, lipoproteins or extracellular RNA, that are typically co-isolated by commonly used isolation methods such as ultracentrifugation (275). On the other hand, other methods that include elimination of concomitant impurities may cause significant reduction of EV yield, which is an important hindrance in terms of the medical use of EVs, where high amounts of EV preparations are required (276). Additionally, recent findings have demonstrated that the “protein corona” which surrounds EVs may be also needed for their biological activity and its removal by additional steps of EV purification may not be beneficial (277). Nevertheless, isolation method is one of the crucial factors that may influence functional properties of EVs and affect their downstream applications.

Another important difficulties to be overcome is a rapid macrophage-dependent clearance of EVs from the circulation (278) and their off-target biodistribution that lowers the level of EV accumulation in the site of interest (36). There are several factors influencing distribution of EVs after their *in vivo* uptake, including route of administration, dosing, cell source (279) and the size of EVs (280), that should be taken under the consideration during the design of EV-related studies.

Moreover, one of the critical bottlenecks in the clinical application of EVs is a lack of unified protocols of their isolation and characterization. Thus, there is also an urgent need for the development of reliable standarization and validation approaches, that would implement rigorous, complementary characterisation methods and would assure no batch-to-batch variation (271). However, due to the extreme complexity and variety of EV-related biological systems, it seems to be a huge challenge to find an optimal and universal experimental layout. As an example, based on the worldwide survey, there are several different isolation methods with ultracentrifugation being the most commonly used. However, the choice of EV isolation method will also vary depending on a type of the starting material, compromise between the purity and yield of obtained EV preparations, as well as their downstream application (281). Another difficulty is a standardized and controlled long-term storage of EV preparations, that would also allow to preserve their biological activity after thawing (282).

One of the critical hallmarks is also a scale-up production, that would not only ensure the sufficient quantity of EVs produced in a good manufacturing practice (GMP) standards, but would also not affect their quality (283). Several groups work on the development of bioreactor-based approaches for the bulk EV production (284). Additionally, scientists try to modify culture conditions of the donor cells, stimulating them physically or chemically in order to significantly increase the yield of secreted EVs (285). Despite existing challenges, several methodological approaches fulfilling GMP standard requirements were reported so far, including e.g. preparation of EVs from BM-MSCs (286) or UC-MSCs (287).

#### 3.3.2 Clinical trials

Despite several encountered difficulties to be overcome to facilitate common use of EVs in the tissue regeneration, the promising results of preclinical studies have become the basis for the attempts on using EV preparations in a medical practice. Currently, there are several clinical trials conducted around the world with the use of EV preparations (288). According to the ClinicalTrials.gov website, on October 2022 there were 84 interventional clinical trials for “extracellular vesicles” inquiry, with 15 of them being already completed. Among the top ones, 25 studies were related to the respiratory tract diseases, 16 to graft versus host disease (GvHD) and 10 to CNS diseases, with majority of them being related to the biomarker studies. Still, the clinical use of EVs for the therapeutic purposes is limited to ongoing early-phase studies, but initial results indicate no significant side effects following EVs administration, indicating their safety and therapeutic potential (289). As an example, in a recently reported case study, EVs derived from UC-MSCs were used for the intracochlear administration in the 55-year old patient suffering from Menière’s disease, who required an insertion of a cochlear implant, that typically causes inflammatory response and local fibrosis that may lead to the hearing loss. Obtained results demonstrated safety of EV injection, attenuation of inflammation and improvement of hearing capacity and speech perception parameter (290). Promising results have led to the preparation of the phase 1 clinical study. In another report, based on the previous data, including those obtained for the nonrandomized open-label cohort study related to the effect of EVs from BM-MSCs in COVID-19 associated ARDS treatment (251), randomized phase 2 clinical study “EXIT-COVID19” has been also conducted, but without already published results. Several other trials are still on the “recruiting” or “not yet recruiting” stage. Thus, direct indication on the effectiveness of EVs in the clinical practice should be expected within the upcoming years, which will allow not only to confirm safety of EV administration, but also to compare efficacy of EVs with the currently available treatments. Based on that it will be possible to indicate the most promising areas of EV-based therapeutic applications as alternatives to the currently utilized approaches.

## 4 Conclusions

Last two decades have brought a significant advancement in the field of EV biology and their potential biomedical utilization. In this review, we have highlighted the recent knowledge on the understanding of the biological activity of EVs, especially those secreted by different types of SCs, in cell-to cell crosstalk, including their role in the regulation of the immune system. In this context, EVs have been widely reported as potential therapeutic factors exhibiting immunoregulatory and pro-regenerative properties. Discovery that EVs may harbour and transfer their bioactive content into the target cells, influencing their fate, opened a new possibilities of use of EV preparations as acellular therapeutic option in several diseases with the inflammatory background. However, despite the vast potential of EVs as drug-delivery systems, their wide utilization is associated with several challenges and limitations that have still to be addressed. Nevertheless, EVs offer a great promise as new-generation tools for an improved diagnostic and clinical purposes.

## Author contributions

EK performed the literature search and wrote the manuscript. PD prepared figures and tables. EKZ-S revised the manuscript. All authors contributed to the article and approved the submitted version.

## Glossary

**Table:**

ACE2	angiotensin converting enzyme 2
AD	Alzheimer’s disease
AFM	atomic force microscopy
AKI	acute kidney injury
ALI	acute lung injury
APCs	antigen presenting cells
AT-MSCs	adipose tissue-derived mesenchymal stem/stromal cells
ARDS	acute respiratory distress syndrome
BBB	blood–brain barrier
BM-MSCs	bone marrow-derived mesenchymal stem/stromal cells
ECM	extracellular matrix
CKD	chronic kidney disease
CM	conditioned medium
CNS	central nervous system
COVID-19	coronavirus infectious disease 2019
CVDs	cardiovascular diseases
CPCs	cardiac progenitor cells
DCs	dendritic cells
EPCs	endothelial progenitor cells
EVs	extracellular vesicles
ESCRT	endosomal sorting complex responsible for transport
ESCs	embryonic stem cells
GMP	good manufacturing practice
GvHD	graft-versus host disease
HCC	hepatocellular carcinoma
HLA	human leukocyte antigen
IBD	inflammatory bowel disease
ILC2s	group 2 innate lymphoid cells
iPSCs	induced pluripotent stem cells
I/R	ischemia/reperfusion
ISEV	International Society for Extracellular Vesicles
LVEF	left ventricular ejection fraction
SCs	stem cells
MHC	major histocompatibility complex
MVBs	multivesicular bodies
MS	multiple sclerosis
MSCs	mesenchymal stem/stromal cells
NTA	nanoparticle tracking analysis
OA	osteoarthritis
PD	Parkinson’s disease
ROS	reactive oxygen species
SNARE	SNAP (soluble NSF attachment protein) receptor
TGF-β	transforming growth factor beta
TNF-α	tumor necrosis factor alpha
UC-MSCs	umbilical cord Wharton’s jelly MSCs
WHO	world health organization.
